# Transcriptomic analysis of paternal behaviors in prairie voles

**DOI:** 10.1186/s12864-022-08912-y

**Published:** 2022-10-01

**Authors:** Florian Duclot, Yan Liu, Samantha K. Saland, Zuoxin Wang, Mohamed Kabbaj

**Affiliations:** 1 Department of Biomedical Sciences, Florida State University, Tallahassee, FL USA; 2 Program in Neuroscience, Florida State University, Tallahassee, FL USA; 3 Department of Psychology, Florida State University, Tallahassee, FL USA

**Keywords:** Alloparenting, Parental care, Medial preoptic area, Nucleus Accumbens, Lateral septum, RNA-sequencing, Mitochondria, RNA translation

## Abstract

**Background:**

The importance of fathers’ engagement in care and its critical role in the offspring’s cognitive and emotional development is now well established. Yet, little is known on the underlying neurobiology due to the lack of appropriate animal models. In the socially monogamous and bi-parental prairie vole (*Microtus ochrogaster*), while 60–80% of virgin males show spontaneous paternal behaviors (Paternal), others display pup-directed aggression (Attackers). Here we took advantage of this phenotypic dichotomy and used RNA-sequencing in three important brain areas to characterize gene expression associated with paternal behaviors of Paternal males and compare it to experienced Fathers and Mothers.

**Results:**

While Paternal males displayed the same range and extent of paternal behaviors as experienced Fathers, we observed structure-specific transcriptomic differences between parental behaviors phenotypes. Using differential expression, gene set expression, as well as co-expression network analyses, we found that phenotypic differences between Paternal males and Attackers were mainly reflected by the lateral septum (LS), and to a lower extent, the nucleus accumbens (NAc), transcriptomes. In the medial preoptic area (MPOA), the profiles of gene expression mainly reflected differences between females and males regardless of their parental behaviors phenotype. Functional enrichment analyses of those gene sets associated with Paternal males or Attackers in the LS and the NAc revealed the involvement of processes related to the mitochondria, RNA translation, protein degradation processes, as well as epigenetic regulation of gene expression.

**Conclusions:**

By leveraging the natural phenotypic differences in parental behaviors in virgin male prairie voles alongside fathers and mothers, we identified a marked structure- and phenotype-specific pattern of gene expression associated with spontaneous paternal behaviors independently from fatherhood and pair-bonding. The LS transcriptome related to the mitochondria, RNA translation, and protein degradation processes was thus highlighted as a primary candidate associated with the spontaneous display of paternal behaviors. Altogether, our observations further characterize the behavioral and transcriptomic signature of parental behaviors in the socially monogamous prairie vole and lay the groundwork to further our understanding of the molecular underpinnings of paternal behavior.

**Supplementary Information:**

The online version contains supplementary material available at 10.1186/s12864-022-08912-y.

## Background

It is now well established that fathers’ contribution in parental care is critical for the well-being of the offspring. The absence of fathers or their low engagement in raising children, are associated with low performance in school, and represent risk factors for numerous behavioral problems and the development of mental health issues such as anxiety, depression, and substance use [1–5]. Accordingly, fathers’ engagement in parenting represents an important target for therapeutic intervention [6, 7] but surprisingly, little is known on the neurobiology of paternal behaviors.

Our current understanding of the neurobiological underpinnings of parental care mainly results from the study of maternal behaviors in mice and rats and highlights the involvement of a coordinated set of structures of a socio-sexual brain network that includes among others the medial preoptic area (MPOA) and the lateral septum (LS) [8–10]. Unlike maternal care, however, paternal care is rare in mammalian rodents, but evidence suggests that the neurocircuitry underlying paternal care shares some similarities with the neurocircuitry of maternal care [11, 12]. To better understand the neurobiology of paternal behaviors, it is important to note that bi-parental care co-occurs with social monogamy in a small subset of mammals [13–15]. Indeed, in socially monogamous species such as the prairie vole (*Microtus ochrogaster*), mandarin vole (*Lasiopodomys mandarinus*), or the California mouse (*Peromyscus californicus*), for instance, fathers are as involved in parental care as mothers, with the exception of nursing [16–21]. These have thus emerged as particularly valuable models to understand the neurobiology of paternal behaviors and brought some evidence for the involvement of a variety of factors and brain structures in paternal care. In line with its role in mice and rats [9], the medial preoptic area (MPOA) is a critical regulator of maternal and paternal behaviors in mothers and fathers in socially monogamous rodents. In prairie voles, fatherhood reprograms gene expression related to neuroplasticity in the MPOA [22], while paternal experience activates neuronal activity in the MPOA [23]. Moreover, California mouse fathers, which show high levels of paternal behaviors, also display higher aromatase activity in the MPOA than virgin males, which have low levels of paternal behaviors [24], whereas MPOA lesion reduces parental behaviors in both mothers and fathers [25, 26]. Interestingly, while electrolytic lesion of the Nucleus Accumbens (NAc) only mildly reduces parental behaviors in California mouse fathers [26], the dopaminergic and oxytocinergic transmissions in the NAc of prairie and mandarin vole fathers are important regulators of parental behaviors. Indeed, fatherhood alters the expression of the oxytocin receptor (OTR) and dopamine D2 receptor (D2R) in the mandarin vole NAc [27], where oxytocin and dopamine releases are activated during bouts of paternal care [28]. Similarly, in pair-bonded male prairie voles, exposure to pups induces dopamine release in the NAc, whereas intra-NAc dopamine D1 receptor (D1R) antagonism reduces the expression of paternal behaviors in pair-bonded males [29]. Altogether, these findings highlight the importance of these structures of the socio-sexual (MPOA) and socio-motivational (NAc) brain networks in the neurobiology of paternal care in fathers.

In prairie voles, in addition to fathers, virgin males naturally display high levels of paternal behaviors [30–32] involving, in part, the LS and NAc. Exposure to pups increases neuronal activity in the Lateral Septum (LS) of virgin male prairie voles [23], where arginine-vasopressin (AVP) neurotransmission through its V1a receptor (V1aR) mediates the expression of paternal behaviors [33]. Similarly, as in pair-bonded males, exposure to pups induces dopamine release in the NAc of virgin males [29]. Notably, not all virgin male prairie voles are highly paternal. While 60–80% of them exhibit spontaneous paternal behaviors towards pups, others display aggressive behaviors [30–32, 34]. These individual differences thus provide a unique opportunity to study the neurobiology of paternal behavior apart from the other, often intrinsically related social behaviors associated with mating, pair-bonding, or fatherhood, which are known to influence the expression of paternal behaviors in prairie voles [29, 34]. The molecular underpinnings driving such individual differences however, remain largely unknown.

In this study, we thus aimed at identifying the molecular signature associated with spontaneous paternal behaviors by comparing the transcriptional profiles of spontaneously alloparental or aggressive adult virgin male prairie voles. To this end, we first characterized the paternal behaviors displayed by spontaneously alloparental virgin male prairie voles against those displayed by actual fathers and mothers. Then, we conducted an unbiased characterization by RNA sequencing of the transcriptomic profile in the MPOA, NAc, and LS based on their repeated involvement in parental behaviors in prairie voles, as well as in other bi-parental and non-parental rodents. To further dissect the molecular regulations associated with paternal behaviors from those related to parenthood or pair-bonding, the transcriptomic signatures of spontaneously paternal or aggressive males were compared to those of mothers and fathers in all three structures. We thus highlight a marked structure- and phenotype-specific pattern of gene expression underlying paternal behaviors in prairie voles suggesting the involvement of the mitochondria, RNA translation, and protein degradation processes. Notably, this gene expression pattern was partially distinct from those underlying fatherhood indicating that despite similar paternal behaviors between Paternal males and experienced fathers, the molecular underpinnings of paternal behaviors differ, at least in part, from fatherhood.

## Results

### Phenotypic characterization of paternal behaviors

While the display of spontaneous paternal behaviors in virgin male prairie voles is well established [30–32, 34], the extent to which these compare to fathers or mothers remains unclear. Before investigating the molecular underpinnings of paternal behaviors in virgin prairie voles, we thus sought to first characterize such spontaneous paternal behaviors and identify those males exhibiting spontaneous paternal behaviors (Paternal group) from those displaying pup-directed aggression (Attackers group). To this aim, adult sexually-naive male prairie voles were first screened in a parental behavior test, alongside fathers and mothers at postpartum day 3.

**Fig. 1:**
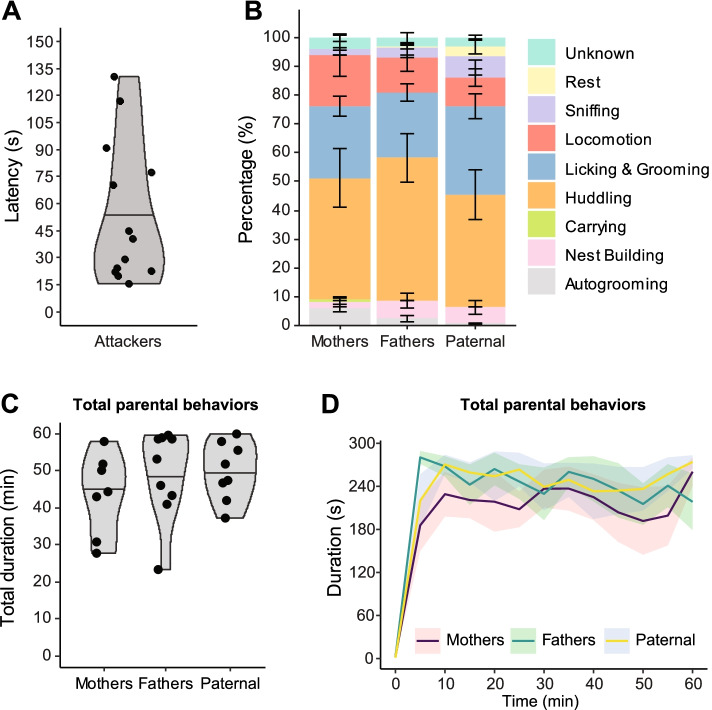
Behavioral performances during the parental behavior test. **A** Latency to display aggressive behaviors in the Attackers group. **B** Distribution of time spent in each of the scored behaviors. **C** Total time spent in total parental behaviors, calculated as the sum of licking & grooming, huddling, nest building, carrying, and sniffing behaviors. **D** Time spent in total parental behaviors across the entire parental behavior test by 5-min timebins. In (**B**, **C**), each data point represents a distinct animal and the shaded area represents the mirrored density estimate of the data points’ distribution; the horizontal line within depicts the 50% quantile of the density estimate. In (**A**, **D**), data is presented as mean ± s.e.m

In line with previous reports, 67.3% of sexually-naive males in our study spontaneously showed paternal behaviors when exposed to an unfamiliar pup, whereas others displayed aggression towards the pup (Fig. 1A, B; median latency to aggression: 40.3 sec, *n* = 13 Attackers). Notably, males from the Paternal group (*n* = 8) showed levels of parental behaviors similar to Fathers (*n* = 9) and Mothers (*n* = 7). Indeed, while Fathers, Mothers, and spontaneously paternal males spent most of the test huddling with the pup, licking & grooming the pup, and to a lower extent in the non-parental behavior “Locomotion” (Fig. 1B), no difference was observed in the time spent exhibiting these behaviors between phenotypes (Additional files 1 and 2). Interestingly, however, while all three groups showed relatively low levels of other parental (nest building, carrying, and sniffing) and non-parental (autogrooming) behaviors, Mothers spent more time autogrooming and carrying the pup than Fathers or Paternal males (Fig. 1B, Additional files 1 and 2). The total time spent in parental behavior nevertheless remained similar between all groups (Fig. 1C, Additional file 2), and evolved similarly across the test (Fig. 1D, F_5.211,109.4_ = 22.8, *p* < 0.0001 for Time, F_2,21_ = 0.77, *p* = 0.475 for Group, and F_24,252_ = 0.62, *p* = 0.917 for the interaction). Moreover, neither the median nor the mean duration of each behavioral bout differed between all three groups (Additional files 3, 4 and 5), indicating that the overall pattern of behaviors displayed upon exposure to an unfamiliar pup did not differ between Paternal males, Fathers, and Mothers.

To highlight potential relationships between the behaviors scored during the parental behavior test, a linear model was fit for each pair of behavior regardless of the phenotype (Mothers, Fathers, and Paternal males). A significant fit was thus found for only 5 out of 36 pairs, indicating that most of the behaviors displayed during the test, parental or not, are not likely to affect other behaviors (Additional file 6). Interestingly, however, a negative link was found between the two most predominant parental behaviors Huddling and Licking & Grooming. Although it could be explained by the mutually-exclusive nature of the behavioral scoring, this observation provides an intriguing parallel with the previously reported individual differences in active vs passive parental care in prairie voles [35]. Nevertheless, to compare the relationships between Licking & Grooming and Huddling for each group, we used linear regression and found no significant interaction for either group (F_2,18_ = 0.71, *p* = 0.507, β = − 0.279 in Mothers, β = − 0.154 in Fathers, and β = − 0.360 in Paternal males). The relationship between Huddling and Licking & Grooming is thus independent on the caregiver type, in line with the lack of group differences in these two behaviors (Additional files 1 and 2). We did, however, found 7 out 72 linear regressions between behaviors showing a significant interaction with the test subject’s group (Additional file 7). The comparisons of slopes for these linear regressions revealed that at the exception of the relationship between the Sniffing and Carry behaviors, all group differences resided between Mothers and Fathers/Paternal males (Additional file 8), indicating that the sex of the caregiver does affect to a moderate extent the relationships between behaviors displayed during the parental behavior test.

Altogether, these observations confirm the previously established phenotypic dichotomy of response upon exposure to an unfamiliar pup in virgin males, with some exhibiting paternal behaviors, while others behaving aggressively towards the pup. Notably, while these observations also confirm previous reports that virgin male prairie voles display the same range of behaviors as experienced fathers, here we further find that the levels of paternal behaviors performed by paternal virgin males are similar to postpartum day 3 fathers in their nature, extent, and timing.

### Structure differences exceed phenotypic differences

**Fig. 2:**
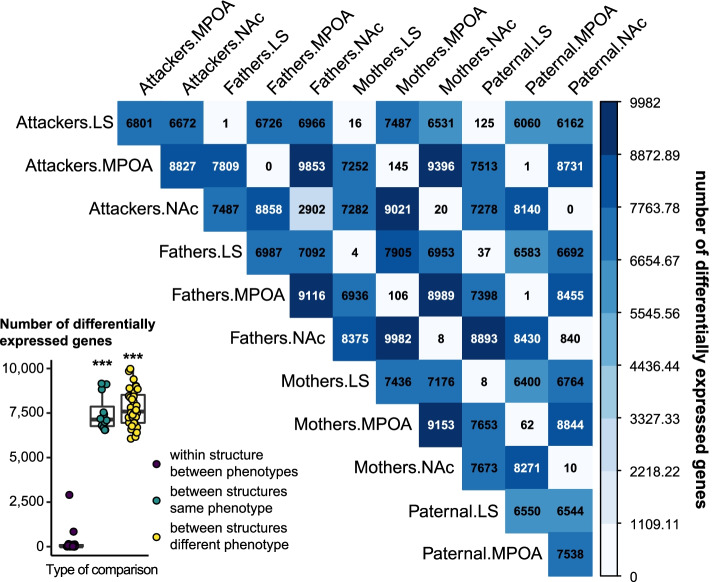
Differential expression analysis across structures. The number of differentially expressed genes is displayed in each pairwise comparison and color-coded according to the scale on the right side of the fig. LS: lateral septum, MPOA: medial preoptic area, NAc: nucleus accumbens

To provide an overview of the transcriptomic regulations associated with parental behaviors across brain structures of interest in prairie voles, we first analyzed differential gene expression across all phenotypes (Mothers, Fathers, Paternal, Attackers) and brain structures (MPOA, NAc, LS). We found a large extent of differential gene expression across all phenotypes between any pair of structures, with the number of differentially expressed genes between structures regardless of the phenotype vastly exceeding the number of differentially expressed genes between phenotypes within a given structure (F_2,63_ = 405.7, *p* < 0.001, *η*^2^ = 0.93, Fig. 2 insert). In paternal males, for instance, 7538 genes were differentially expressed (DE) between the MPOA and the NAc, 6550 between the MPOA and the LS, and 6544 between the NAc and LS (Fig. 2). The gene ontologies and pathways associated with these DE genes were highly overlapping between phenotypes and enriched for a wide array of processes related to neurotransmission, signal transduction, and synaptic plasticity (Additional file 9), thereby illustrating the extent of transcriptomic differences between brain structures related to neuroplasticity in prairie voles.

### Structure-specific differential expression between phenotypes

Such extensive and overlapping differences in gene expression across phenotypes, however, are likely to mask differences within structures and thus impede the analysis of differences between phenotypes within structures. As a result, we then repeated the differential expression analysis between phenotypes within each structure and found a marked structure-specific pattern of differential expression between phenotypes (Additional files 10, 11 and 12).

**Fig. 3:**
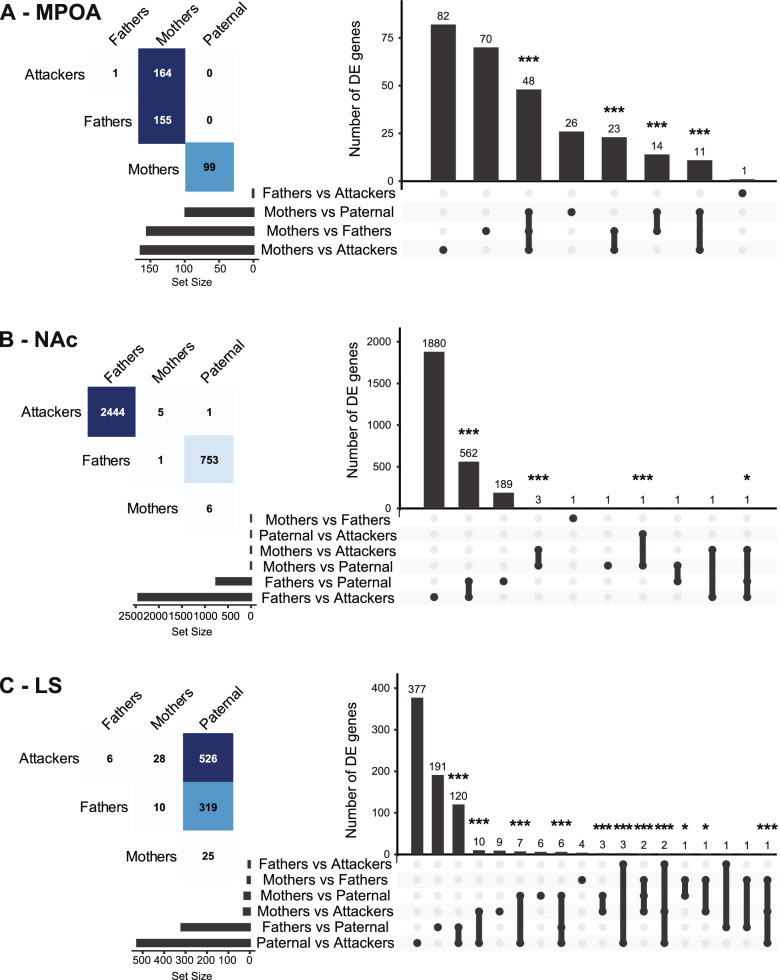
Differential expression analysis within structures. The number of differentially expressed (DE) genes (left) and their overlap (right) is displayed for each pairwise comparison in the medial preoptic area (MPOA, **A**), nucleus accumbens (NAc, **B**), and lateral septum (LS, **C**). On the right, UpSet plots [36, 37] depict the intersections between each set (pairwise comparison) of differentially expressed (DE) genes as matrix of dots with each row corresponding to a set and each column corresponding to a possible intersection. Dots are colored in grey when the given set (row) is not included in the given intersection (column), or in black when it is included. Joined black dots depict the overlap between given sets. For instance, a total of 164 genes were found DE between Mothers and Attackers in the MPOA (A, left). Among these 164 DE genes, 82 are unique to the Mothers vs Attackers comparison, 23 are also found DE in the Mothers vs Fathers comparison, 11 are also found DE in the Mothers vs Paternal comparison, and 48 are also found DE in the Mothers vs Paternal as well as Mothers vs Fathers comparisons (A, right). *p < 0.05, ***p < 0.001, hypergeometric test for overlaps between two or three sets of genes

In the MPOA, we found moderate differential expression between Mothers and Fathers (155 DE genes), Attackers (164 DE genes), or Paternal males (99 DE genes), while only 1 or 0 genes were DE between any of the male groups (Fathers, Paternal, or Attackers, Fig. 3A). Notably, DE genes between Mothers and any of the male groups were substantially overlapping (31.0% vs. Fathers, 48.5% vs. Paternal, and 29.3% vs. Attackers), indicating that patterns of differential gene expression in the MPOA mainly reflect differences between female and male prairie voles regardless of their cohabitation status or parental behaviors phenotype. Accordingly, the gene ontologies associated with these sexually-biased genes were enriched for terms related to sexual differentiation, general signaling pathways, and synapse (Additional files 13, 14 and 15), suggesting sex differences in neuroplasticity in the prairie vole MPOA. Such sex differences cannot solely be explained by sex chromosomes, however, as DE genes were distributed throughout the genome (Additional file 16).

**Fig. 4:**
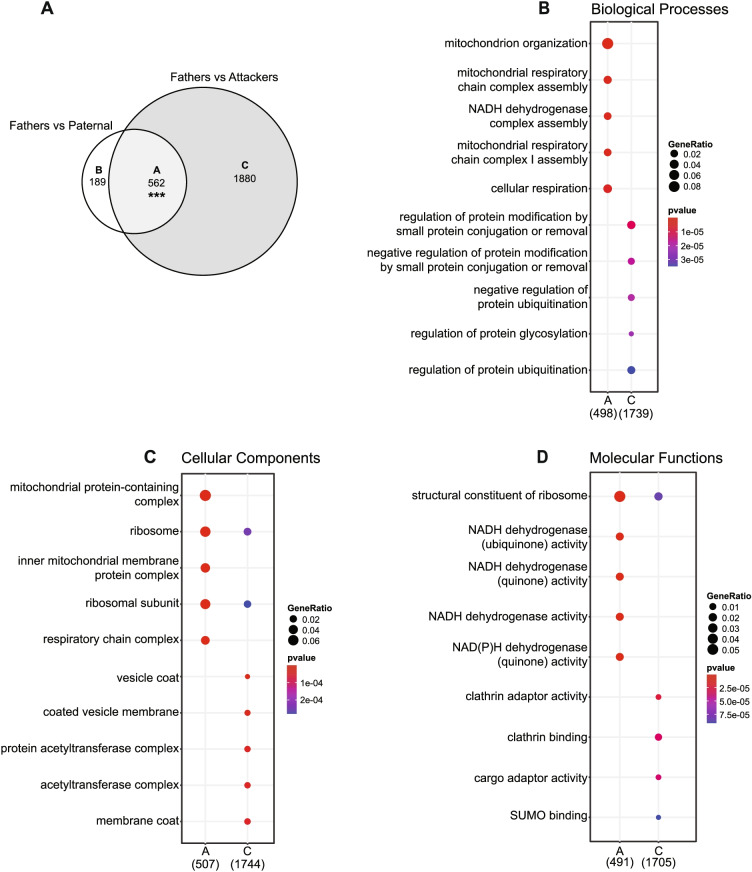
Functional enrichment of overlapping and distinct sets of differentially expressed genes in the nucleus accumbens (NAc). In (**A**), the number of differentially expressed genes overlapping or distinct between fathers and virgin males in the NAc is depicted. For each set, the functional enrichments in gene ontologies of the biological processes (**B**), cellular components (**C**), and molecular functions (**D**) categories are displayed. ***p < 0.001, hypergeometric test for overlap between two sets of genes

In the NAc, we found high levels of differential gene expression in Fathers when compared to Paternal males (753 DE genes), or Attackers (2444 DE genes), while only 1 to 6 DE genes were detected in any other comparison (Fig. 3B). This, therefore, suggests that the patterns of gene expression in the NAc within males reflect adaptations related to cohabitation and pair-bonding more than those associated with parental behavior. Nevertheless, it is interesting to note that while 74.6% of DE genes found in Fathers vs. Paternal males overlap with those vs. Attackers, only 23% of DE genes between Fathers and Attackers are also DE between Fathers and Paternal males, thereby indicating that the majority of changes in Attackers when compared to Fathers is distinct from those in Paternal males. As a result, even though only 1 gene reached the statistical threshold for differential expression between Paternal males and Attackers (Fig. 3B), the transcriptional profile in the NAc of a sexually-naive male could in part depend on its alloparental behaviors phenotype. In line with the overlap in DE genes between the Fathers vs. Paternal and Fathers vs. Attackers comparisons, the gene ontologies and pathways enriched in both of these gene sets overlap as well and relate to mitochondria, synapse, RNA translation, and signal transduction (Additional file 17). The consideration of specific subsets of genes nevertheless reveals distinct functional profiles between phenotypes. Indeed, while the 562 DE genes in common between Fathers and virgin males (Paternal and Attackers) are highly enriched in terms related to mitochondrial function, those found DE only between Fathers and Paternal males relate to mitochondria and RNA translation, whereas those DE between Fathers and Attackers relate to protein degradation and turnover, epigenetic regulation of gene expression through histone acetylation, and the synapse (Fig. 4, Additional file 14). In addition to further supporting our previous findings on the enrichment of mitochondrial function and RNA translation in the NAc following pair-bonding in prairie voles [38], these observations thus suggest that these processes might also be involved to a lower extent in the regulation of paternal behaviors.

Contrary to the MPOA and the NAc, we found substantial differential gene expression within virgin males in the LS, as Paternal males exhibit 526 DE genes when compared to Attackers, and 319 DE genes when compared to Fathers. A relatively small number of DE genes was found in any of the other comparisons (Fig. 3C), suggesting that most of the transcriptomic regulations in the LS reflect differences in paternal behaviors phenotype. Moreover, 71.6% of DE genes in Paternal males when compared to Attackers are distinct from those DE when compared to Fathers, which further highlights that the profile of differential transcriptional regulation between Paternal males and Attackers is related to their parental behaviors phenotype. On the other hand, a separate subset of 191 genes was DE between Fathers and Paternal males but not within virgin males, thereby indicating that the LS transcriptome does reflect, at least in part, the effect of fatherhood. Notably, none of these genes were also DE in Fathers when compared to Attackers, which distances these genes from the paternal behaviors phenotype, and thus indirectly further strengthens their association to fatherhood. Nevertheless, one cannot rule out the possibility that the molecular correlates of paternal behaviors in the LS differ between virgin males and Fathers despite seemingly similar behavioral performances. In this context, it is interesting to note the substantial overlap in differential gene expression in Paternal males when compared to Attackers and Fathers, as this subset of genes could be considered as such molecular underpinning for a fatherhood-dependent control of paternal behavior.

**Fig. 5:**
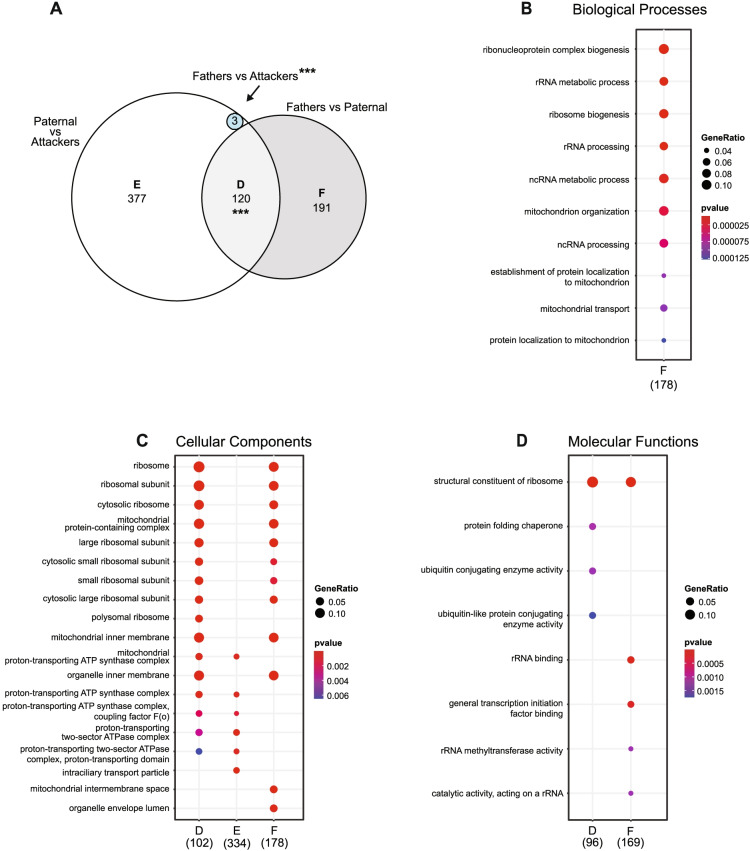
Functional enrichment of overlapping and distinct sets of differentially expressed genes in the lateral septum (LS). In (**A**), the number of differentially expressed genes overlapping or distinct within males in the LS is depicted. For each set, the functional enrichments in gene ontologies of the biological processes (**B**), cellular components (**C**), and molecular functions (**D**) categories are displayed. ***p < 0.001, hypergeometric test for overlaps between two sets of genes

Conversely, although only 3 genes were DE in Fathers when compared to Attackers (*Slc2a5*, *Sgk1*, and *Col16a1*), these overlapped with DE genes within virgin males, but not between Paternal males and Fathers (Fig. 3C), thereby highlighting a small set of genes associated with paternal behaviors regardless of fatherhood status. In this context, the enrichment of gene ontologies and pathways associated with the genes DE in Paternal males when compared to Attackers or Fathers suggests an involvement of RNA processing & ribosomes, mitochondrial functions, and cilia (Additional file 18), in the spontaneous expression of paternal behaviors in sexually naive male prairie voles. Such involvement of mitochondrial function was particularly illustrated by the enrichment of terms related to the ATP synthase complex in the genes DE only between paternal males and attackers (Fig. 5), thereby further detailing a key candidate process in the LS for the spontaneous expression of paternal behaviors in prairie voles. Genes DE between Fathers and paternal males, on the other hand, were highly enriched in terms related to ribosomes and RNA translation, and to a lower extent mitochondria (Fig. 5). This was interestingly the case in those specific to this group (set “F” in Fig. 5), or common with the Paternal vs Attackers comparisons (set “D” in Fig. 5), associating these processes in the LS with fatherhood in interaction or not with the spontaneous display of paternal behaviors--although via distinct subsets of genes.

To explore the source of the transcriptomic regulations we observed, we proceeded with a cell-type deconvolution analysis by leveraging a publicly-available single-cell RNA-seq dataset identifying cell-type specific gene expression in the mouse ventral striatum [39]. We thus found that neurons and astrocytes were the two main cell types detected in our dataset across all three structures (Additional file 19A). When considering DE genes, however, we only detected the neuronal cell type in the MPOA and LS, while both the astrocyte and neuron cell types were observed in the NAc (Additional file 19B). Interestingly, the estimated proportion of the neuronal cell type in the LS was greater in paternal males than in Attackers or Fathers, but not in the MPOA or the NAc (Additional files 19C and 20). Although the single-cell reference dataset originates from a different species and structure, these observations nevertheless suggest that neurons are the main candidate cell type in which the differential gene expression associated with paternal males occurs. Notably, given an acute exposure to the pup, such as occurring during the parental behavior test, is known to increase c-Fos expression in several brain areas including the MPOA and LS in male prairie voles [23, 40, 41], we sought to describe the extent to which immediate early genes (IEG) such as c-Fos are represented in the DE genes in our dataset. In all three structures analyzed in our study, we found very few to no IEG in DE genes in any comparison (Additional file 21), indicating that the acute effect of pup exposure on the transcriptomic patterns we observed remain limited.

Altogether, these observations denote structure-specific associations with the parental behaviors phenotype. Indeed, while the profile of gene expression in the MPOA mainly reflects differences between males and females, the transcriptomic profiles in the NAc were mainly associated with the cohabitation and pair-bonding status and to a lower extent to the parental behaviors phenotype in males. Differences in gene expression were highly associated with the male parental behaviors phenotype in the LS, however, denoting this structure as a particularly interesting area underlying individual differences in paternal behaviors in sexually-naive male prairie voles.

### Structure-specific functional enrichment

Although our differential expression analysis brought valuable insight into the structure- and phenotype-specific profiles of gene expression related to the spontaneous display of paternal behaviors in prairie voles, this approach is restricted to the use of a statistical significance threshold and thus could inaccurately exclude small but widespread differences in gene expression. To provide an unbiased functional understanding of the transcriptomic patterns underlying differences between groups, we thus conducted a threshold-free gene set enrichment analysis (GSEA) within each structure and then visualized the resulting gene sets using an enrichment map depicting the directionality of change for each regulation.

**Fig. 6:**
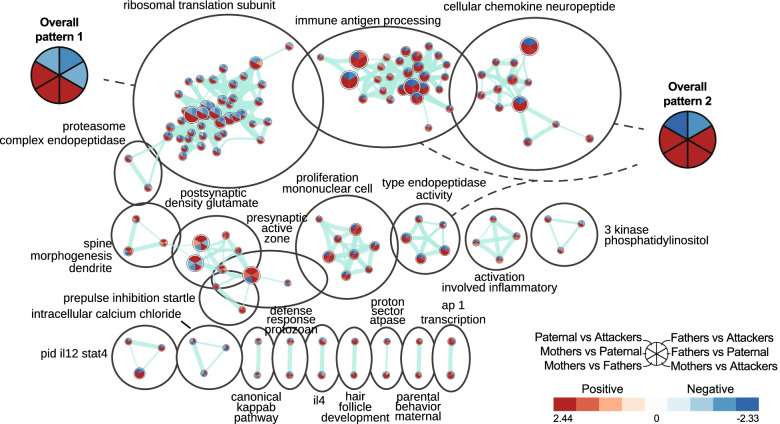
Gene sets enrichments in the medial preoptic area (MPOA). Enrichment map depicting the clusters of differentially modulated pathways between phenotypes in the MPOA identified by gene set enrichment analyses. The area of each node, representing a gene set (functional pathway), corresponds to the number of genes of the gene set it contains, and its color depicts the direction of enrichment (red: positive, blue: negative) with the color intensity representing the enrichment score in the given pairwise comparison (see in-figure legend). Edge thickness is proportional to the number of genes overlapping between the two connected nodes. “Overall patterns” summarize the common profile of enrichment within given clusters of gene sets. Grey sectors within these summaries depict enrichments variable between gene sets of the given clusters. Ap 1: activator protein 1; il4: interleukin 4; pid il12 stat4: interleukin 12 (IL12) signaling mediated by signal transducer and activator of transcription 4 (STAT4)

In the MPOA, we observed an enrichment of gene sets consistent with the differential expression analysis as most clusters such as those related to translation and the immune system exhibit the same directionality in comparisons involving mothers, thereby depicting a sex bias unrelated to the parental behaviors phenotype (overall patterns 1 and 2, Fig. 6). A group of clusters related to the synapse shows a phenotype-dependent sex difference, however, with a bias towards Mothers when compared to Attackers, but no clear bias when compared to other male groups. Interestingly, a group of clusters related to the immune system and chemokine signaling shows a phenotype-dependent regulation within males, with an opposite profile of regulation between Fathers and either virgin male groups: up-regulated when compared to Paternal, down-regulated when compared to Attackers (overall pattern 2, Fig. 6). As a result, although our data further support our previous observation that sex differences are the main contributor to transcriptomic variability in the MPOA, they do suggest the involvement of genes related to the immune system and chemokine signaling in the expression of spontaneous paternal behaviors in prairie voles.

**Fig. 7:**
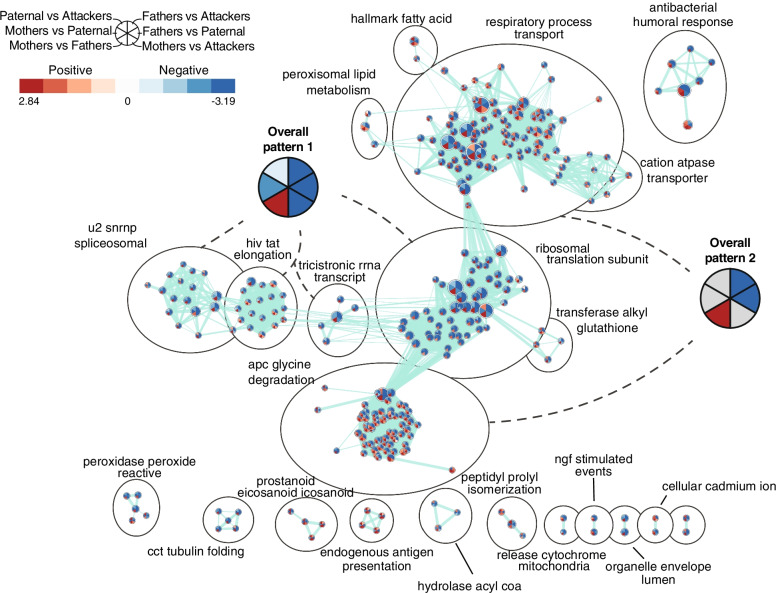
Gene sets enrichments in the nucleus accumbens (NAc). Enrichment map depicting the clusters of differentially modulated pathways between phenotypes in the NAc identified by gene set enrichment analyses. The area of each node, representing a gene set (functional pathway), corresponds to the number of genes of the gene set it contains, and its color depicts the direction of enrichment (red: positive, blue: negative) with the color intensity representing the enrichment score in the given pairwise comparison (see in-figure legend). Edge thickness is proportional to the number of genes overlapping between the two connected nodes. “Overall patterns” summarize the common profile of enrichment within given clusters of gene sets. Grey sectors within these summaries depict enrichments variable between gene sets of the given clusters. Apc: anaphase-promoting complex; cct: chaperonin-containing TCP-1; hiv: human immunodeficiency virus; ngf: nerve growth factor; u2 snrnp: U2 small nuclear ribonucleoprotein complex

In the NAc, we found a widespread enrichment of gene sets related to processes such as mitochondrial function, RNA translation, RNA splicing, and protein degradation (Fig. 7). Notably, the direction of enrichment for all these clusters remained generally similar within males, regardless of their fatherhood status or parental behaviors phenotype (overall patterns 1 and 2, Fig. 7), supporting the results from differential expression analysis and further confirming the involvement of these processes in pair-bonding [38]. Interestingly, all significantly enriched gene sets were found biased towards Mothers when compared to Fathers, which thus further illustrates the sexually-biased nature of the NAc transcriptome in pair-bonded prairie voles [38, 42].

**Fig. 8:**
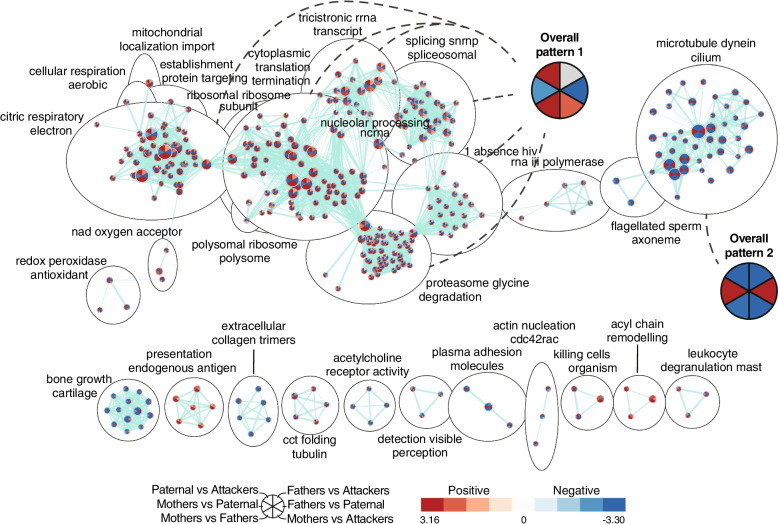
Gene sets enrichments in the lateral septum (LS). Enrichment map depicting the clusters of differentially modulated pathways between phenotypes in the LS identified by gene set enrichment analyses. The area of each node, representing a gene set (functional pathway), corresponds to the number of genes of the gene set it contains, and its color depicts the direction of enrichment (red: positive, blue: negative) with the color intensity representing the enrichment score in the given pairwise comparison (see in-figure legend). Edge thickness is proportional to the number of genes overlapping between the two connected nodes. “Overall patterns” summarize the common profile of enrichment within given clusters of gene sets. Grey sectors within these summaries depict enrichments variable between gene sets of the given clusters. Hiv: human immunodeficiency virus; nad: nicotinamide adenine dinucleotide; snrnp: small nuclear ribonucleoprotein complex

In the LS, we also found a widespread enrichment of gene sets related to mitochondrial function, RNA translation, and protein degradation (Fig. 8). In line with our differential expression analysis, we found a high enrichment of these gene sets biased towards paternal males when compared to Attackers and Fathers, but not between Fathers and Attackers (overall pattern 1, Fig. 8), thereby strengthening the association of these gene sets with the spontaneously parental nature of males in the Paternal group. Unlike in our differential expression analysis, however, the GSEA reveals that the directionality of enrichment throughout gene sets in the LS followed a sex bias dependent on the male parental phenotype (father, paternal, or attacker). Indeed, while the Mothers vs Fathers and Mothers vs Attackers comparisons present with the same bias across gene sets within a given cluster (overall patterns 1 and 2, Fig. 8), this bias is reversed when mothers are compared to paternal males. Altogether, this evidence supports the fact that the transcriptome in the LS of spontaneously paternal males is distinct from other male phenotypes.

### Structure-specific gene networks underlying parental behaviors phenotype

To relate the transcriptomic patterns described above to each individual’s parental behaviors phenotype, we conducted a weighted gene co-expression network analysis (WGCNA) to highlight clusters of genes with similar expression (modules) and extract modules of interest based on their relation to the behavioral performance during the parental behavior test.

**Fig. 9:**
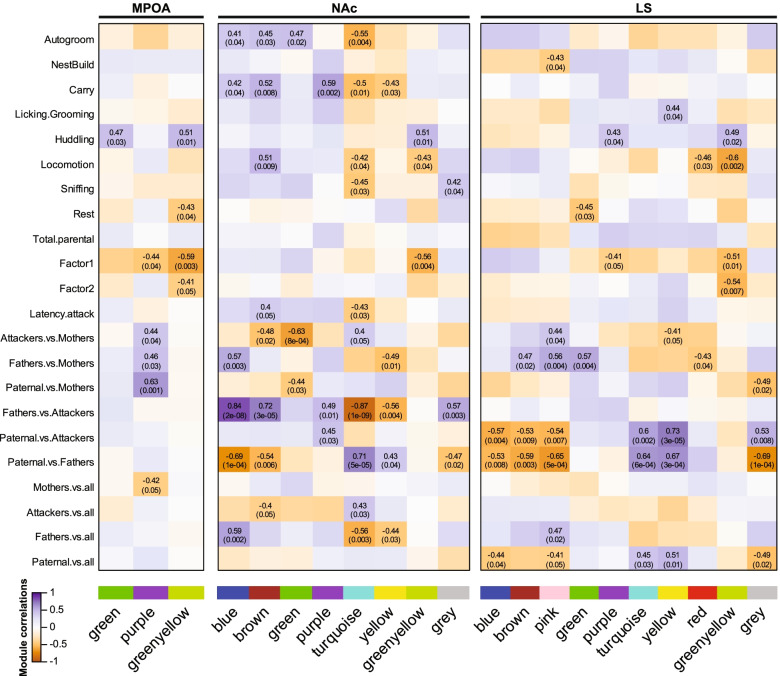
Structure-specific associations of gene coexpression modules with parental behaviors. The correlation of each co-expression module from the weighted gene coexpression network analysis with behavioral traits (behaviors scored during parental behavior test and phenotype status) is depicted for each structure. For all significant associations (*p* ≤ 0.05), the correlation value is detailed alongside its corresponding *p*-value in parentheses. Note that within each structure, only modules with at least one significant association are depicted. The correlation and *p*-values for all associations are shown in Additional File 22. MPOA: medial preoptic area, NAc: nucleus accumbens, LS: lateral septum

We thus identified 10 consensus modules of gene expression with substantial levels of varying correlation with behavioral traits across structures. Indeed, while only 3 modules were found significantly associated with at least one trait in the MPOA, 8 and 10 modules were characterized as such in the NAc and LS, respectively (Fig. 9, Additional file 22). This therefore further supports the greater suggested involvement of the LS, and NAc to a lower extent, than the MPOA in the behavioral differences in parental behaviors in virgin males. In further accordance with our differential expression analysis, the purple module was found linked to the Mothers phenotype in the MPOA but not in the NAc or LS. Interestingly, genes from the purple module relate to oligodendrocytes and myelination processes (Additional file 23), which might thus underline sex differences in myelination in the MPOA between prairie vole mothers and fathers. We did, however, find the green and greenyellow modules correlated with the Huddling, Factor 1, and Factor 2 traits, suggesting some level of association between select members of the MPOA transcriptome and parental behaviors.

Additional structure-specific associations were similarly found in the NAc and LS. In the NAc, for instance, the blue, brown, and green to a lower extent, modules are positively associated with the Fathers phenotype, especially when compared to Attackers as well as behaviors implicating movement such as Locomotion and Carry. In the LS, however, this green module’s association diverges from the blue and brown modules, whereas the pink module here shows a similar profile. Moreover, the association with this group of modules with the Fathers phenotype as well as the Locomotion and Carry behaviors is lower in the LS than in the NAc, whereas its link with the Paternal phenotype is greater, especially when compared to Attackers. Similarly, the turquoise and yellow group of modules is negatively associated with the Fathers phenotype in the NAc especially when compared to virgin males, regardless of their parental behaviors phenotype, as well as with the Locomotion, Carry, Autogrooming, and Sniffing behaviors (Fig. 9). In the LS, however, this group of modules is joined by the red module—which has a similar profile of associations, although to a lower extent—but here exhibits a marked positive association with the Paternal phenotype, including when compared to other male groups. Interestingly, while most of the associations of this group of modules with behaviors found in the NAc do not hold in the LS, we found a significant positive correlation between the yellow module and the licking & grooming behavior. A functional analysis of the genes comprising the blue, brown, and pink modules revealed their relation with the establishment of synapse and synaptic signal transduction, those from the green module with protein degradation, whereas genes from the turquoise, yellow, and red modules relate to the ribosome, RNA translation, metabolism, and the mitochondria, (Additional files 23, 24, 25, 26 and 27).

Altogether, these observations further support the greater association of the LS and the NAc with the parental behaviors phenotype of virgin male prairie voles when compared to the MPOA, whose significant modules of interest preferentially reflect sex differences in the MPOA. Despite some degree of similarity between the modules’ associations with behavioral traits in the NAc and the LS, substantial structure-dependent differences were detected in modules related to synaptic transmission, RNA translation, and mitochondrial function.

## Discussion

In this study, we investigated the transcriptomic underpinnings of paternal behaviors in the socially monogamous prairie vole. We first characterized and screened for male prairie voles that would display spontaneous behaviors upon exposure to a pup and reproduced the previously described dichotomy in response of virgin males [30–32, 34] as some spontaneously presented with paternal behaviors, whereas others exhibited pup-directed aggression. Interestingly, we found that Paternal males displayed the same range, nature, and extent of parental behaviors than Fathers and Mothers. Despite such behavioral similarities, however, Paternal males presented with a distinct transcriptomic profile in the brain when compared to Fathers, Mothers, and Attacker males. These differences, however, were highly structure-specific. While the profiles of gene expression in the MPOA mainly reflected differences between females and males regardless of their parental behaviors phenotype, the LS transcriptome, as well as the NAc transcriptome to a lower extent, were associated with differences in paternal behaviors in virgin males. Furthermore, these structure- and phenotype-specific patterns of gene expression highlight the involvement of the mitochondria, RNA translation, and protein degradation in the neuroadaptations underlying the expression of spontaneous paternal behaviors in sexually naive male prairie voles.

We first reproduced the established individual differences in parental behaviors displayed by adult virgin male prairie voles. Indeed, while some males spontaneously cared for the unfamiliar pup, others quickly showed signs of aggression towards the pup (median latency to aggression: 40.3 sec). Interestingly, when compared to Fathers with previous parental care experience, not only did these paternal males show the same range of parental behaviors, they were indistinguishable in their total duration, their individual bouts durations, as well as their occurrence over the test session. This observation is in accordance with recent reports describing the lack of differences in parental behaviors between virgin male prairie voles and experienced fathers, including after 6 weeks of fatherhood experience [32, 43]. Nevertheless, an early study did report a progressive increase in the levels of paternal behaviors following cohabitation and mating with a female resulting in higher levels of paternal behaviors in experienced fathers at postpartum day 6 when compared to virgin males [34]. Although this discrepancy could be explained by the difference in fatherhood experience (postpartum day 6 vs postpartum day 3 fathers used in our study), it could also result from variations in behavioral testing paradigms or environmental factors such as early life experiences, known to alter prairie voles’ parental care phenotype [35, 44, 45]—a hypothesis that would deserve further examination. Altogether, this nonetheless indicates that in addition to being amongst the few mammalian species to display paternal care, the prairie vole represents a unique opportunity to study the neurobiology of paternal care in disconnection from known interactions with sexual experience, pair-bonding, and fatherhood.

To get insight into the global molecular signature underlying spontaneous paternal behavior in male prairie voles, we analyzed their transcriptomic profile in three brain structures associated with parental behaviors in prairie voles and other rodents by RNA sequencing. Notably, we leveraged the unique opportunity offered by the spontaneous nature of paternal behaviors in virgin males by comparing their transcriptomic profile to virgin males displaying pup-directed aggression, experienced fathers, as well as mothers, which allowed the discrimination of the gene expression patterns associated with paternal behaviors from those associated with fatherhood, sexual experience, and pair-bonding. Moreover, this analysis was conducted in the MPOA, NAc, and LS, three brain areas implicated in parental behaviors in prairie voles, mice, and rats, to delineate each structure’s role. In line with the high heterogeneity in gene expression between large anatomical areas in the rodent brain [46], the differences between structures in our dataset far outweigh those between phenotypes and highlight variations in multiple aspects of neuroplasticity between structures. Considering each brain area separately, however, revealed a structure-specific association of the transcriptomic signature and phenotype.

The anatomical sexual dimorphism of the MPOA is well established in mice and rats [47, 48], but is more complex in socially monogamous species such as prairie voles. Indeed, although early reports describe similar volume, cell number, and cell density between males and females [49], the density of vasopressin (AVP) fibers in the MPOA is greater in males than females [34], highlighting the presence of sexual dimorphism in the prairie vole MPOA. In line with such observation, we found moderate sex differences at the transcriptomic level in the MPOA revealed by a sexually biased differential expression and a consistent directionality of gene set enrichment in Mothers when compared to any group of males, regardless of their parental behaviors phenotype or fatherhood status. In comparison, we only found limited evidence for a link between the transcriptomic signature of the MPOA and spontaneous paternal behaviors. Due to the nature of tissue collection used in our study, we cannot rule out the possibility that some of the hypothalamic nuclei surrounding the MPOA might be represented in our dataset, thereby potentially diluting differences between sexually-naïve males. Nevertheless, even though no gene was found DE within virgin males, we did observe a slight interaction between the prominent sex differences in the MPOA and the paternal phenotype, as illustrated by a small cluster of gene sets related to the synapse that shows a marked bias towards Mothers when compared to Attackers, but not when compared to other groups of males. Interestingly, such involvement of genes related to the synapse in the MPOA has previously been associated with differences between fathers and virgin males in prairie voles [22], along with genes linked to neurotransmission or immune functions. Notably, while we did reproduce such involvement of genes related to immune functions when comparing fathers to virgin males, we show here that such transcriptomic underpinnings of fatherhood can be dependent on the parental behaviors phenotype of virgin males. Clusters of gene sets related to the immune system and chemokine signaling indeed showed an opposite profile of regulation in Fathers when compared to Paternal males, than when compared to Attackers. In light of the known role of the immune system and microglia in the establishment of sex differences in the brain, including the MPOA, and the regulation of social behaviors in various rodents including prairie voles [50–55], it is particularly interesting to consider these processes as potential modulators of paternal behaviors in fathers, for instance. Furthermore, in light of the critical role of the MPOA in the regulation of maternal care [9, 12, 56], it is important to note that due to the absence of a group of alloparental females in our study, it remains unknown whether these transcriptomic patterns associated with parental behaviors in males would be similar in females.

In the NAc, most of the group differences in differential expression, gene sets enrichment, or association of co-expression modules with traits were between Fathers and virgin males, thus mainly reflecting the transcriptomic correlates of pair-bonding we previously reported [38]. Interestingly, these effects of pair-bonding on the NAc transcriptome greatly differ between sexes [38] and are thus likely to explain the limited differential expression observed between virgin males and Mothers. The NAc transcriptome nonetheless showed some level of association with the alloparental behavior of virgin males, however, as the differences between virgin males and Fathers were partly dependent on their parental behaviors phenotype. The biological pathways and processes associated with these genes relate to protein degradation and turnover, epigenetic regulation of gene expression through histone acetylation, and the synapse, which would thus suggest the involvement of sustained adaptations of neurotransmission in the NAc in the expression of paternal behaviors. Accordingly, blockade of the D1R-mediated neurotransmission in the prairie vole NAc reduces the expression of paternal behaviors in pair-bonded males [29]. Such involvement of the neurotransmission in the NAc is interestingly also observed in the socially monogamous mandarin voles, in which DA is released in the NAc during bouts of paternal care in fathers, alongside an enhanced oxytocin neurotransmission [28]. Although we did not find significant differences in *Drd1a* or *Oxtr* genes levels in the NAc, we did observe an enrichment of biological processes related to signal transduction in the NAc in the genes up-regulated in Fathers when compared to Attackers. This enrichment was absent in the DE genes between Fathers and Paternal males, however, which therefore further suggests that such association with neurotransmission in the NAc relates to parental behaviors of Fathers rather than their inherent pair-bonded status or sexual experience.

In contrast to the relatively limited evidence linking the MPOA transcriptome to spontaneous paternal behaviors in our study, the profiles of gene expression in the LS exhibited strong associations with the paternal behaviors phenotype. Indeed, unlike the MPOA and the NAc, the LS showed substantial differential expression between Paternal males and Attackers and a marked association of co-expression modules with the Paternal phenotype. Moreover, this profile of differential expression as well as the widespread enrichment of gene sets observed in Paternal males was distinct from those detected against Fathers, or between Fathers and Attackers. In addition to further supporting the specificity of this pattern of gene expression to the spontaneously paternal phenotype in virgin males, this underscores a molecular substrate for the dichotomy in paternal behaviors displayed by virgin male prairie voles. Our analysis also revealed that the LS transcriptome reflects a complex interaction between the spontaneous display of paternal behaviors in virgin males, and the effects of fatherhood. Indeed, while most of the differences were specific to Paternal males, a subset of genes remained specific to the Fatherhood, whereas a distinct set of genes appeared related to the paternal phenotype in a fatherhood-dependent manner. This would thus suggest that while some transcriptional patterns are linked to the spontaneous paternal phenotype but not to fatherhood, others are underlying paternal behaviors in fathers. In other words, these observations would suggest that the mechanisms underlying paternal behaviors in virgin males and in fathers are both distinct from each other and overlapping.

In accordance with a preponderant role for the LS in paternal behaviors in virgin males, local AVP injection promotes the display of paternal behaviors in sexually-naive male prairie voles, an effect prevented by V1aR antagonism [33]. AVP in the LS also facilitates the formation of partner preference, however [57], which illustrates how neuroadaptations controlling paternal behaviors can overlap with those underlying social bonding. Similarly, it remains unclear whether the transcriptomic differences between Paternal males and Attackers in the LS truly reflect the molecular substrate of paternal behaviors, or rather those directing aggression. Indeed, aggression in male prairie voles is associated with neuronal activation in the LS [58] and, following up on the same example as above, AVP neurotransmission in the LS can also affect aggression levels in rats and mice [59, 60]. Such overlap notably affects a greater range of socially motivated behaviors as, for instance, variations in V1aR expression in the LS are linked to social approach in prairie voles [61], whereas V1aR blockade in the LS increases social play in juvenile male rats [62]. In this context, it would be interesting to consider the extent to which the LS transcriptome reflects such a balance of socially motivated behaviors like paternal care or pup-directed aggression. The set of genes specific to Paternal males when compared to Attackers interestingly provides evidence for an involvement of mitochondrial function and ATP synthase in particular, in modulating such balance.

Screening virgin male prairie voles was a necessary step in our study to characterize their behavioral profile and to determine their paternal behaviors phenotype. It is however important to note that an acute exposure to a pup triggers by itself a variety of neuroendocrine and physiological responses in prairie voles, as well as neuronal activation in several brain areas including the MPOA and LS [23, 32, 40, 41]. In particular, 3 hrs of pup exposure increase the expression of the IEG Fos in the medial amygdala, MPOA, medial bed nucleus of the stria terminalis (BNST), LS, and several thalamic nuclei in males [23]. Shorter exposures can also lead to IEG activation as a 30-min or a 20-min exposure increases the number of Fos-positive cells in the medial amygdala and BNST when measured 90 mins after its initiation [41], or in the paraventricular nucleus of the hypothalamus when measured 1 hr. after its initiation [40], respectively. In our dataset, we only found very few to no IEG represented in the DE genes across the MPOA, NAc, and LS (Additional file 21), suggesting that the effect of acute pup exposure on the transcriptomic signatures we identified remained limited. Although the contribution of such acute effects cannot be ruled out, this observation would thus suggest that the main transcriptomic patterns we highlighted in our study are pre-existent to the parental behavior test. It thus appears that variations in a collection of genes in key brain areas such as the LS are implicated in the ontology of the dichotomy in parental behaviors found within virgin male prairie voles, similar to how variations in gene expression in the brain are associated with characteristic behaviors of monogamous species, including parental care, when compared non-monogamous counterparts [63–71].

Interestingly, early life experiences are likely key contributing factors to these variations and parental care itself represents a critical component. Early life adverse events such as paternal deprivation or physical stress can lead in adulthood to neuroadaptations in brain areas including among others the MPOA, NAc, and LS, alongside impairments in social behaviors characteristic of social monogamy such as pair-bonding and alloparenting [72–78]. Moreover, prairie vole parents vary in their amount and type of parental care, which in turn has long term consequences on the offspring’s social behaviors in adulthood. In particular, the offspring receiving high levels of parental care in turn display higher levels of alloparental behaviors than the offspring from low parental care breeders [35]. Notably, this effect is dependent on the behavior of the rearing parents rather than genetic parents [44], which highlights a critical and long-term influence of the offspring’s early environment on its social abilities later in life. In this case, it becomes particularly interesting to consider epigenetic mechanisms as they provide a prime interface between one’s environment and enduring changes in gene expression, and have been increasingly implicated in various aspects of prairie voles’ behaviors [79–83]. In particular, low levels of paternal care lead to DNA methylation of the MT2 region of the *oxtr* gene and lower OTR mRNA levels in the NAc [84, 85]. Interestingly, DNA methylation of the MT2 region in whole blood samples correlates with OTR expression in the prairie vole NAc [84], and occurs in a region conserved between prairie voles and humans [85], thereby presenting with qualities for a use as a biomarker and for studying humans. Similarly, paternal deprivation increases DNA methylation in the 3′-UTR of the *Avpr1a* gene in the LS, resulting in higher V1aR mRNA levels [61]. In our study, it is thus particularly interesting to note the enrichment of processes related to the epigenetic regulation of gene expression via histone acetylation in the genes differentially expressed between Fathers and Attackers in the NAc. Altogether, although the link between such epigenetic modifications and alloparental behaviors remains to be demonstrated, these observations do highlight how epigenetic mechanisms in the NAc and LS could participate to establish the transcriptomic patterns related to the display of spontaneous paternal behaviors in prairie voles that we uncovered.

## Conclusions

In this study, we aimed at identifying the molecular signature associated with spontaneous paternal behaviors by leveraging the unique phenotypic dichotomy of virgin male prairie voles. While the display of spontaneous paternal behaviors in virgin male prairie voles is well established, the extent to which these compare to fathers or mothers remained unclear. Here, we found that the range and characteristics of paternal behaviors exhibited by paternal virgin males are indistinguishable from those displayed by fathers, indicating that paternal virgin male prairie voles can be considered as a relevant model to study the neurobiology of paternal behaviors representative of paternal behaviors displayed by fathers, yet without the potential confounding effects of fatherhood and pair-bonding. Despite such behavioral similarities, we uncovered a highly structure-specific pattern of transcriptomic regulation associated with the spontaneous display of paternal behaviors and identified its overlap or distinction from the transcriptomic regulations associated with fatherhood or pair-bonding. Indeed, the display of paternal behaviors emerged associated primarily with the LS and to a lower extent the NAc transcriptomes, whereas the molecular signature associated with fatherhood was primarily reflected by the NAc transcriptome. Nevertheless, we found that the LS transcriptome also recapitulates a complex interaction between the spontaneous display of paternal behaviors in virgin males and the effects of fatherhood, thereby suggesting that the mechanisms underlying paternal behaviors in virgin males and in fathers are both distinct from each other and overlapping. The profiles of gene expression in the MPOA, however, mainly reflected differences between females and males regardless of their parental behaviors phenotype. These structure- and phenotype-specific patterns of gene expression highlight the involvement of the mitochondria, RNA translation, and protein degradation in the neuroadaptations underlying the expression of spontaneous paternal behaviors in virgin male prairie voles. It is important to note, however, that the direct and causal relationship between these transcriptomic patterns and paternal behaviors remain to be tested, and that the main key driver genes remain to be established. Altogether, our observations further characterize the behavioral and transcriptomic signature of parental behaviors in the socially monogamous prairie vole and lay the groundwork to further our understanding of the molecular underpinnings of paternal behavior.

## Methods

### Animals and experimental design

Male and female prairie voles (*Microtus ochrogaster*) from a laboratory breeding colony were weaned at 21 days of age and housed in same-sex sibling pairs in plastic cages (12 × 28 × 16 cm) with water and food provided ad libitum. All cages were maintained under a 14:10 h light-dark cycle, and the temperature was approximately 20 °C. All animals were randomly assigned into experimental groups and used for paternal behavior testing at 231 ± 8.3 days. This test allowed for the classification of sexually-naive males into Paternal or Attackers based on the display of paternal or aggressive behaviors, respectively (see below). To better characterize the paternal behaviors displayed by spontaneously alloparental virgin male prairie voles, we compared their behavioral performance to experienced fathers and mothers. To do so, a separate group of intact adult prairie voles was paired and cohabitated to allow for pregnancy. These voles were thus first-time parents and constitute the Fathers and Mothers groups and were tested in the parental behavior test at 3 days postpartum. As most sexually-naïve males were tested for their paternal behaviors towards pups at postnatal day 3, voles in the Fathers and Mothers groups were tested at postpartum day 3 to reduce potential variations from the pup-associated stimulus. Experimental procedures were approved by the Institutional Animal Care and Use Committee at Florida State University.

### Parental behaviors test

The parental behavior test was conducted similarly to previously described [29, 72] and based on an established protocol [86, 87]. All test subjects (Mothers, Fathers, Paternal, Attackers) were exposed to the same protocol described below. First, the test subject was placed in a Plexiglas cage (20 × 25 × 45 cm) larger than its home cage, containing clean bedding, water, and food. Following a 15-min habituation period, a 1–3 day old unfamiliar stimulus pup was introduced in the corner of the testing cage opposite to the test subject, and the test subject was allowed to freely interact with the pup for 60 mins. The 60-min session was video-recorded and the following behaviors of the test subject were scored a priori by a trained experimenter blind to the treatment groups using JWatcher (v1.0) [88]. Sexually-naïve prairie voles display direct caregiving behaviors (known as alloparenting) including carrying (pup-retrieval), huddling, licking & grooming, and sniffing the pup as well as nest building, and these behaviors have been extensively characterized and studied in previous studies [32, 72, 89]. Notably, about 60% sexually-naïve male prairie voles are spontaneously alloparental whereas others either attack or ignore the pup [90, 91]. Further, alloparental behaviors displayed by sexually-naïve male prairie voles are no different from paternal behaviors displayed by actual fathers [92–94]. In the present study, we quantified each pattern of the above-mentioned alloparental behaviors. We also scored subject’s autogrooming, resting away from the pup, and locomotion. All other behaviors were scored as “unknown”. As most sexually-naïve males were tested for their paternal behaviors towards pups at postnatal day 3, voles in the Fathers and Mothers groups were tested at postpartum day 3 to reduce potential variations from the pup-associated stimulus. If the sexually-naive male test subject attacked the pup, the experimenter tapped the cage to immediately stop the behavioral testing, the pup was removed from the testing cage and the test subject was classified as Attacker, as described previously [31, 34]. As the behavioral testing was stopped at the first attack, no other behavior was scored for the Attackers.

### RNA extraction, library preparation, and sequencing

RNA extraction was conducted as previously described [38]. Immediately after the parental behavior test, subjects were killed by rapid decapitation, their brain dissected out, snap-frozen, and stored at − 80 °C until further processing. Brains were sliced into 200 μm sections on a cryostat and thaw-mounted on slides. Thereafter, 1 mm-diameter punches from 3 (MPOA) or 4 consecutive sections (NAc and LS) were taken from the NAc (Plates 12–18), LS (Plates 19–27), and MPOA (Plates 32–40; Additional file 28) [95]. Total RNA was then extracted from tissue punches taken from 200 μm sections using the TRI-Reagent protocol according to the manufacturer’s instructions (Molecular Research Center, Cincinnati, OH, USA), followed by DNAse I treatment to remove any eventual DNA contamination and clean-up (RNA Clean & Concentrator, Zymo Research, Irvine, CA, USA). RNA quality and integrity were then verified electrophoretically on an RNA Nano 6000 Bioanalyzer chip (Agilent, Santa Clara, CA, USA), whereas RNA concentration was measured spectrophotometrically (Nanodrop, Thermo Fisher Scientific, Waltham, MA, USA).

RNA samples were then sent to the Florida State University NGS Library Facility for the preparation of RNA sequencing (RNA-seq) libraries following poly(A) mRNA purification. A total of 72 barcoded and unstranded RNA-seq libraries were thus generated: *n* = 6 per group (chosen as representative of their respective group based on behavioral results from the parental behaviors test) with 4 groups (Mothers, Fathers, Paternal, and Attackers) and 3 structures each (MPOA, NAc, and LS). All libraries were then pooled and sequenced a first time (2x150bp, NovaSeq 6000, S4 lane), followed by the generation of a second pool of the same libraries then sequenced on the same instrument (2x150bp, NovaSeq 6000, S1 lane) at the Translational Sciences Laboratory at Florida State University. Reads from both sequencing runs were combined, yielding a total of 2899.43 M paired-end raw reads (passing filter, >Q30, and demultiplexed), with a median number of reads per biological sample of 38.85 M. The data discussed in this publication have been deposited in NCBI’s Gene Expression Omnibus [96] and are accessible through GEO Series accession number GSE190213.

### Data processing and differential expression analysis

Raw reads were first processed for quality filtering and adapter trimming with fastp (v0.20.0) [97], followed by verification of good quality using FastQC (v0.11.8) [98] before pseudoalignment and quantification with Salmon (v0.14.1) [99] using 1000 bootstraps and the --validateMappings, −-rangeFactorizationBins 4, −-seqBias, −-gcBias, and --recoverOrphans flags, to improve the sensitivity and specificity of mapping as well as correcting for common systematic biases [99, 100]. Notably, quantification was done using Ensembl annotations (release 97) of the prairie vole genome (MicOch1.0, GCA_000317375.1) to which the *Avpr1a* gene sequence (AF069304) was manually added. Quantifications were thus summarized at the gene level using the tximport R package [101] and then processed for differential expression analysis using DESeq2 (v1.32.0) [102] with design = ~ Group (Group: Mothers/Fathers/Paternal/Attackers). As required by DESeq2, the gene counts estimated by tximport were directly imported in DESeq2 without prior normalization [102]. Libraries normalization, estimate of dispersion, count outliers detection and exclusion, and statistical testing were then conducted using DESeq2’s default settings. Genes with a false discovery rate less of less than 10% were classified as differentially expressed; no threshold based on fold-change was used. As per the authors’ recommendation, an initial inspection of all samples by principal component analysis revealed the presence of three outliers (1 Attacker-LS, 2 Paternal-MPOA) likely resulting from technical processing given all animals used for RNA sequencing were chosen as representative of their respective group. These three outliers were thus excluded from the dataset before all statistical analyses of sequencing data.

### Functional analysis

Gene-Sets Enrichment Analyses (GSEA, v3.0) [103] were performed as previously described [38, 104] using gene-sets comprising pathway annotations for mouse curated from public databases (http://download.baderlab.org, May_01_2020 release), and the resulting enriched pathways were visualized using the Cytoscape (v3.8.2) [105] enrichment map plugin [106], following the author’s recommendations [107]. Following the authors’ recommendations, normalized gene counts exported from DESeq2 were used. Notably, gene annotations in RNAseq matrices were enhanced with known gene orthologues from the mouse genome fetched from Biomart (Ensembl release 97) [108]. To do so, prairie vole Ensembl gene ids without gene symbol were attributed their corresponding gene symbol from mouse orthologs with a “one-to-one” relationship. This procedure was then repeated for those remaining without a gene symbol by using the mouse orthologs with a “one-to-many” relationship; in such case all mouse orthologs for a given prairie vole gene were sorted (in order) based on confidence of homology (high or low), mouse gene-order conservation score, followed by percentage of identity with the query gene, and the best (top) hit was kept. This resulted in an additional 2061 genes annotated for a total of 16,341 genes annotated with a gene symbol out of 17,532 genes present in our dataset. To improve the clarity of enrichment maps, overall patterns of gene-set enrichment were summarized for clusters of interest (Figs. 6, 7 and 8) by calculating the median of the enrichment readout (using the EnrichmentMap::Colouring value) per pairwise comparison for all gene-sets of the cluster of interest. Grey sectors within these overall patterns summaries depict enrichments with variable directionality between gene-sets of the given clusters.

The representation of immediate early genes (IEG) in the differentially expressed (DE) genes was calculated by comparing our lists of DE genes with a published list of curated IEG [109]. For each pairwise comparison in our study, the list of genes overlapping between these two lists were extracted and statistical enrichment was tested using Fisher’s exact test in R [110]. Throughout the study, the enrichment of related gene ontologies and Kyoto Encyclopedia of Genes and Genomes (KEGG) [111–113] pathways was tested using the Bioconductor package clusterProfiler (v4.0.0) [114, 115].

### Gene network analyses

A weighted gene co-expression network analysis was conducted using the R package WGCNA (v1.69) [116–118] following a variance stabilization transformation of the RNA-seq counts as per the authors’ recommendations. Notably, as the extent of between-structures differences in each group (Mothers, Fathers, Paternal, Attackers) was substantial, signed co-expression networks were first constructed within each structure using biweight midcorrelation. This approach thus reduced the number of samples used to construct the topological overlap matrix (MPOA: 22, NAc: 24, LS: 23), which given the sensitivity of WGCNA to very low sample sizes could constitute a limitation in the interpretation of the resulting network of gene co-expression. It is important to note, however, that although the sample size used in our study could reduce the WGCNA’s power, it remains above the minimum of 20 samples recommended by the WGCNA authors [119], thereby justifying the validity of this approach in our study. Following the authors’ instructions, consensus clusters of gene expression (modules) were then extracted, and behavioral traits (behavioral measurements during the parental behavior test) were then related to consensus modules eigengenes within each structure. A factor analysis of the behaviors scored during the parental behavior test revealed two factors (termed Factor 1 and Factor 2) encompassing the main behaviors displayed during the test. The sample loadings for each of these factors were thus included as additional behavioral traits. As per the WGCNA authors’ recommendations regarding the case of categorical variables such as the phenotype in our study (Mothers, Fathers, Paternal, Attackers), binary indicators representing contrasts between two levels of the variable (pairwise comparison, e.g. Paternal.vs. Attackers) or for a level vs. all other levels (e.g. Paternal.vs. All) were created. While each gene was assigned to a single module, we obtained three sets of consensus module eigengenes as a given module might have a particular expression profile in each of the three structures analyzed. While 10 consensus modules were thus identified, the modules most related to our behavioral data (and significant with *p* < 0.05) were extracted by sorting the matrices of biweight midcorrelation according to the following criteria (in order): licking & grooming duration, huddling duration, total parental duration, factor 1, factor 2, nest building duration, as well as binary traits related to group membership Paternal_vs_all, Paternal_vs_Attackers, Paternal_vs_Fathers, and Paternal_vs_Mothers—each considering their *p*-value first, followed by the correlation value. The resulting modules with similar direction and significance to a given behavioral trait were then grouped, and their respective genes tested for functional enrichment in gene ontologies and KEGG pathways using the clusterProfiler R package [114, 115].

### Statistics and reproducibility

Throughout this study, each animal corresponds to a biological replicate and a total of 37 prairie voles were used in the behavioral analysis (Mothers: 7, Fathers: 9, Paternal: 8, Attackers: 13), 24 of which were used for the transcriptomic analysis (Mothers: 6, Fathers: 6, Paternal: 6, Attackers: 6). Data were analyzed with the Prism (v9.1.2, GraphPad Software, San Diego, CA, USA) or R [110] softwares by one-way analysis of variance using “Phenotype” (Mothers, Fathers, Paternal, and Attackers when applicable) as the independent factor, followed by Tukey’s post-hoc test when a main effect was statistically significant at alpha = 0.05. Timeline data presented in Fig. 1D were analyzed using a mixed-effects analysis with Time, Group (Mothers, Fathers, Paternal) and their interaction as fixed effect, and Subject as the random effect. In Figs. 3, 4 and 5, and Additional file 15, the probability of occurrence by chance of the overlap between sets of genes was tested using an hypergeometric test from the R stats package [110] for overlaps between 2 sets of genes, or the R package SuperExactTest [120, 121] for overlaps between 3 sets of genes; an overlap was considered significant when *p* < 0.05.

## Supplementary Information

**Additional file 1.** Time spent in each behavior during the parental behavior test. The total duration across the entire test session is depicted for each animal for the autogrooming, carry, huddling, licking & grooming, locomotion, nest building, and sniffing behaviors; each data point thus represents a distinct animal. The horizontal line in the shaded violin represents the 50% quantile of the density estimate.

**Additional file 2.** Analysis of variance (ANOVA) results for all behaviors scored during the parental behavior test.

**Additional file 3.** Behavioral bouts analysis. For each behavior scored, the duration of each bout was summarized by mean or median across the entire parental behavior test for each animal. Each data point represents a distinct animal, and the boxplots depict the median (thick horizontal line) and the 25th and 75th percentiles.

**Additional file 4.** Depiction of individual behavioral bouts across the parental behavior test. The bouts of the autogroom, carry, huddling, licking & grooming, nest building, and sniffing behaviors are depicted across time throughout the entire parental behavior test session. Each row represents a distinct animal.

**Additional file 5.** Analysis of variance (ANOVA) results for behavioral bouts analysis.

**Additional file 6.** Relationships between parental behavior test behaviors. For each pair of behavior, a linear model was fit. Each individual point represents a distinct animal, and the shaded area depicts the 95% confidence interval.

**Additional file 7.** Detailed results from the linear regressions between all behaviors scored during the parental behaviors test, testing for an interaction of Group (Mothers/Fathers/Paternal).

**Additional file 8.** Comparisons of slopes for linear regressions between behaviors scored during the parental behaviors test. Slopes (a) and their differences between groups (b) are listed for all linear regressions with a significant interaction with Group (see Additional file 7).

**Additional file 9.** Functional enrichment across structures and phenotypes. For each pairwise comparison, the functional enrichments in gene ontologies of the biological processes (BP), cellular components (CC), and molecular functions (MF) categories as well as in pathways from the Kyoto Encyclopedia of Genes and Genomes (KEGG) were tested for the differentially expressed genes up- or down-regulated (UP, and DOWN, respectively). The labels of pairwise comparisons were coded in two letters: the first one represents the phenotype (M: mothers, F: fathers, P: paternal males, A: attackers), whereas the second one represents the structure (N: NAc). For instance, FNvPN refers to the comparison between fathers and paternal males in the NAc.

**Additional file 10.** Differential expression analysis details. For conciseness, the labels of pairwise comparisons were coded in two letters: the first one represents the phenotype (M: mothers, F: fathers, P: paternal males, A: attackers), whereas the second one represents the structure (M: MPOA, N: NAc, L: LS). For instance, PLvAL refers to the comparison between the Paternal and Attackers phenotypes in the LS. DE: differentially expressed.

**Additional file 11.** Summary statistics for the differential expression analysis. DE: differentially expressed. LS: lateral septum, MPOA: medial preoptic area, NAc: nucleus accumbens.

**Additional file 12. **Volcano plots for the differential expression analysis. These plots depict the log2 fold-change (x-axis) against the -log10 of the uncorrected *p*-value (y-axis) for each gene in each pairwise comparison in the medial preoptic area (MPOA, A), nucleus accumbens (NAc, B), and lateral septum (LS, C). Differentially expressed genes are depicted in red.

**Additional file 13.** Functional enrichment of genes differentially expressed in the medial preoptic area (MPOA). For each pairwise comparison, the functional enrichments in gene ontologies of the biological processes (A), cellular components (B), and molecular functions (C) categories were tested for the differentially expressed genes up- or down-regulated (UP, and DOWN, respectively). The labels of pairwise comparisons were coded in two letters: the first one represents the phenotype (M: mothers, F: fathers, P: paternal males, A: attackers), whereas the second one represents the structure (M: MPOA). For instance, MMvFM refers to the comparison between the mothers and fathers in the MPOA.

**Additional file 14.** Functional enrichment of genes differentially expressed in the medial preoptic area (MPOA), the nucleus accumbens (NAc), or the lateral septum (LS). For each pairwise comparison, the functional enrichments in gene ontologies of the biological processes (BP), cellular components (CC), and molecular functions (MF) categories as well as in pathways from the Kyoto Encyclopedia of Genes and Genomes (KEGG) were tested for the differentially expressed genes up- or down-regulated (UP, and DOWN, respectively). The labels of pairwise comparisons were coded in two letters: the first one represents the phenotype (M: mothers, F: fathers, P: paternal males, A: attackers), whereas the second one represents the structure (L: LS). For instance, PLvAL refers to the comparison between the Paternal and Attackers phenotypes in the LS.

**Additional file 15. **Functional enrichment of overlapping and distinct sets of differentially expressed genes in the medial preoptic area (MPOA). In (A), the number of differentially expressed genes overlapping or distinct between all sets of sexually biased comparisons in the MPOA is depicted. For each set, the functional enrichments in gene ontologies of the biological processes (B), cellular components (C), and molecular functions (D) categories are displayed. ***p < 0.001, hypergeometric test for overlaps between two or three sets of genes.

**Additional file 16.** Representation of the number of genes differentially expressed in the medial preoptic area (MPOA, top), nucleus accumbens (NAc, middle), and lateral septum (LS, bottom) across all chromosomes and linkage groups in the prairie vole assembly. Only cases with at least one differentially expressed gene are depicted.

**Additional file 17.** Functional enrichment of genes differentially expressed in the nucleus accumbens (NAc). For each pairwise comparison, the functional enrichments in gene ontologies of the biological processes (A), cellular components (B), and molecular functions (C) categories as well as in pathways from the Kyoto Encyclopedia of Genes and Genomes (KEGG, D) were tested for the differentially expressed genes up- or down-regulated (UP, and DOWN, respectively). The labels of pairwise comparisons were coded in two letters: the first one represents the phenotype (M: mothers, F: fathers, P: paternal males, A: attackers), whereas the second one represents the structure (N: NAc). For instance, FNvPN refers to the comparison between fathers and paternal males in the NAc.

**Additional file 18.** Functional enrichment of genes differentially expressed in the lateral septum (LS). For each pairwise comparison, the functional enrichments in gene ontologies of the biological processes (A), cellular components (B), and molecular functions (C) categories as well as in pathways from the Kyoto Encyclopedia of Genes and Genomes (KEGG, D) were tested for the differentially expressed genes up- or down-regulated (UP, and DOWN, respectively). The labels of pairwise comparisons were coded in two letters: the first one represents the phenotype (M: mothers, F: fathers, P: paternal males, A: attackers), whereas the second one represents the structure (L: LS). For instance, PLvAL refers to the comparison between the Paternal and Attackers phenotypes in the LS.

**Additional file 19.** Estimated cell type proportions. The proportions of various cell types were estimated in our dataset using a publicly-available single-cell RNA sequencing dataset. While panel (A) shows the estimated proportions for all genes detected in our study, panels (B) and (C) depict the estimated proportion of the “Astrocytes” and “Neurons” cell types, or only “Neurons”, respectively, in genes differentially expressed in the given structure.

**Additional file 20.** Analysis of variance (ANOVA) results for the estimated proportions of the “Neurons” cell type in the genes differentially expressed in the medial preoptic area (MPOA), nucleus accumbens (NAc), and lateral septum (LS).)

**Additional file 21.** Number of immediate early genes (IEG) in the genes differentially expressed in the medial preoptic area (MPOA), nucleus accumbens (NAc), and lateral septum (LS).

**Additional file 22. **Structure-specific associations of gene coexpression modules with parental behaviors. The correlation of each co-expression module from the weighted gene coexpression network analysis with behavioral traits (behaviors scored during parental behavior test and phenotype status) is depicted for each structure. The correlation value is detailed alongside its corresponding *p*-value in parentheses. Note that within each structure, only modules with at least one significant association are depicted. MPOA: medial preoptic area, NAc: nucleus accumbens, LS: lateral septum.

**Additional file 23.** Functional enrichment for modules and groups of modules derived from the weighted gene co-expression analysis (WGCNA). For each pairwise group of modules, the functional enrichments in gene ontologies (GO) of the biological processes (BP), cellular components (CC), and molecular functions (MF) categories as well as in pathways from the Kyoto Encyclopedia of Genes and Genomes (KEGG).

**Additional file 24.** Functional enrichment of gene ontologies of the biological processes category in gene co-expression modules derived from the weighted gene coexpression network analysis (WGCNA).

**Additional file 25.** Functional enrichment of gene ontologies of the cellular components category in gene co-expression modules derived from the weighted gene coexpression network analysis (WGCNA).

**Additional file 26.** Functional enrichment of gene ontologies of the molecular functions category in gene co-expression modules derived from the weighted gene coexpression network analysis (WGCNA).

**Additional file 27.** Functional enrichment of pathways from the Kyoto Encyclopedia of Genes and Genomes (KEGG) in gene co-expression modules derived from the weighted gene coexpression network analysis (WGCNA).

**Additional file 28.** Representative location of tissue punches collection. As no atlas in stereotaxic coordinates exists for the prairie vole brain, representative plates from the rat brain atlas [95] are depicted. Tissue punches were taken from sections ranging from plates 12–18 for the nucleus accumbens (NAc, A), plates 19–27 for the lateral septum (LS, B), and plates 32–40 for the medial preoptic area (MPOA, C).

## Data Availability

The datasets generated and analyzed during the current study are available in the NCBI’s Gene Expression Omnibus [96] repository (accession number: GSE190213, https://www.ncbi.nlm.nih.gov/geo/query/acc.cgi?acc=GSE190213).
