# Supplementary figures and images for: Transcriptomic analysis of paternal behaviors in prairie voles
Source: BMC Genomics. 2022 Oct 1;23:679. doi: 10.1186/s12864-022-08912-y (PMC9526941; doi:10.1186/s12864-022-08912-y)

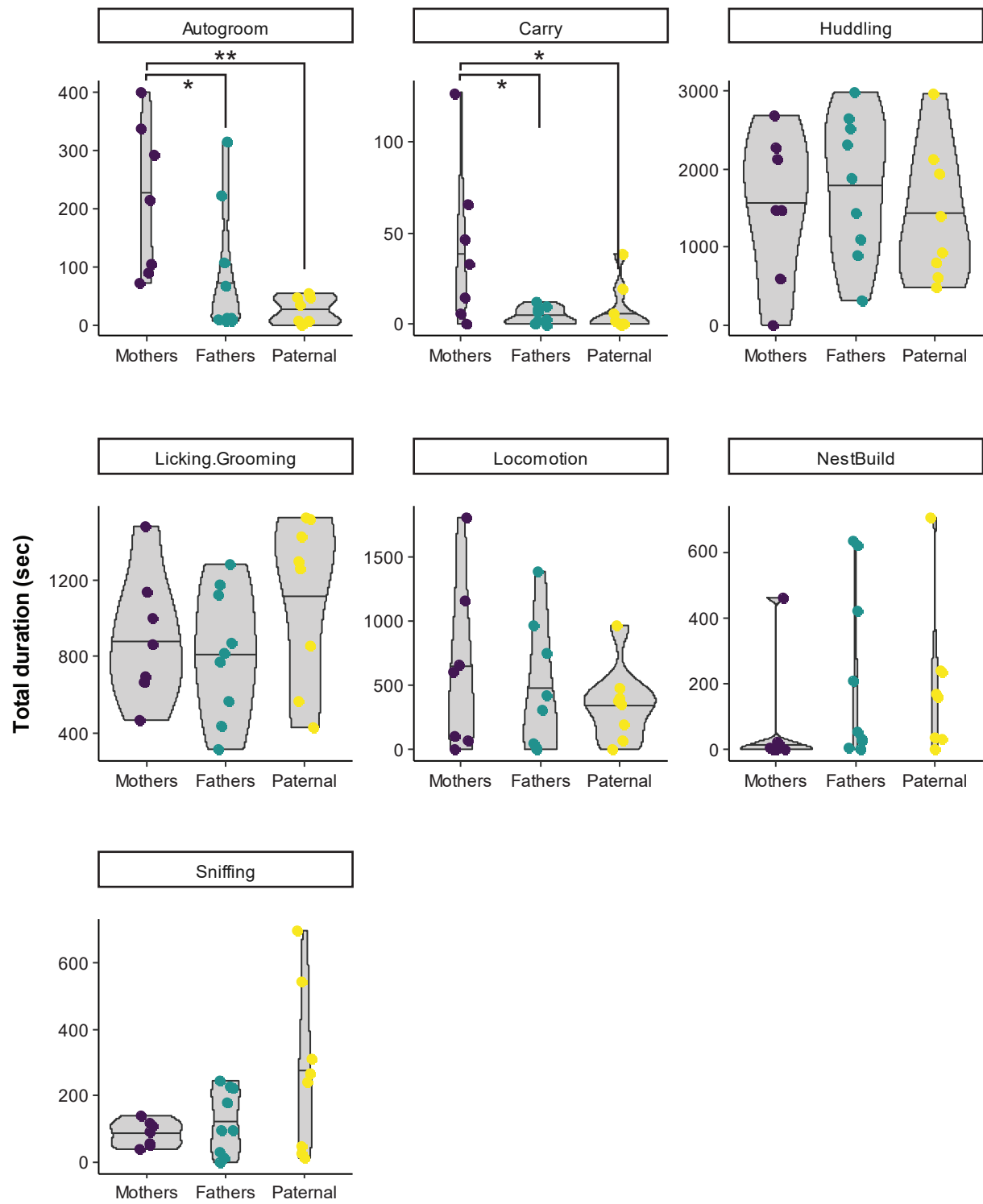

Supplement: Supplementary file 1 — Additional file 1. Time spent in each behavior during the parental behavior test. The total duration across the entire test session is depicted for each animal for the autogrooming, carry, huddling, licking & grooming, locomotion, nest building, and sniffing behaviors; each data point thus represents a distinct animal. The horizontal line in the shaded violin represents the 50% quantile of the density estimate. [file 12864_2022_8912_MOESM1_ESM.pdf]

Mean and Median duration of each bout

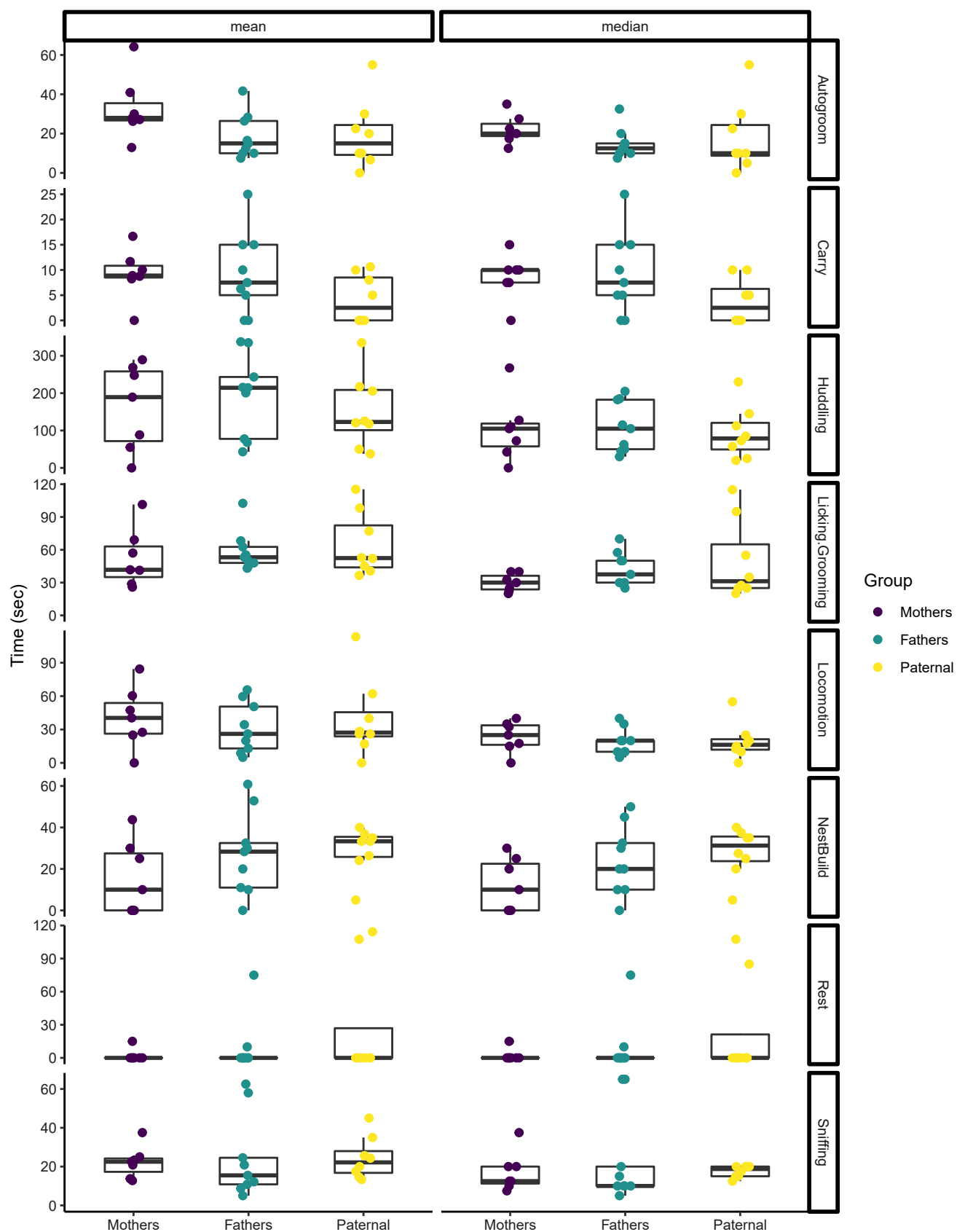

Supplement: Supplementary file 3 — Additional file 3. Behavioral bouts analysis. For each behavior scored, the duration of each bout was summarized by mean or median across the entire parental behavior test for each animal. Each data point represents a distinct animal, and the boxplots depict the median (thick horizontal line) and the 25th and 75th percentiles. [file 12864_2022_8912_MOESM3_ESM.pdf]

## Behavioral bouts

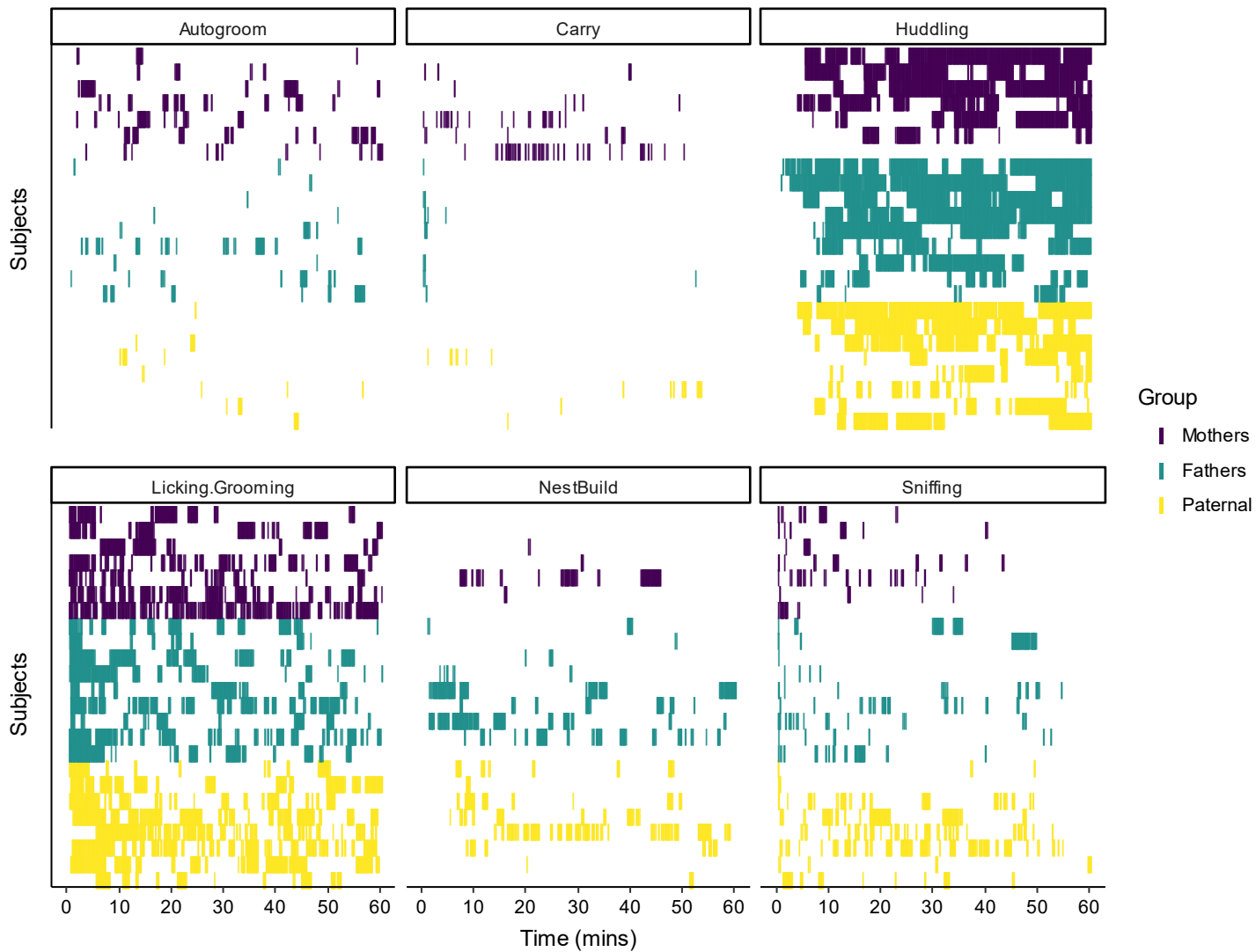

Supplement: Supplementary file 4 — Additional file 4. Depiction of individual behavioral bouts across the parental behavior test. The bouts of the autogroom, carry, huddling, licking & grooming, nest building, and sniffing behaviors are depicted across time throughout the entire parental behavior test session. Each row represents a distinct animal. [file 12864_2022_8912_MOESM4_ESM.pdf]

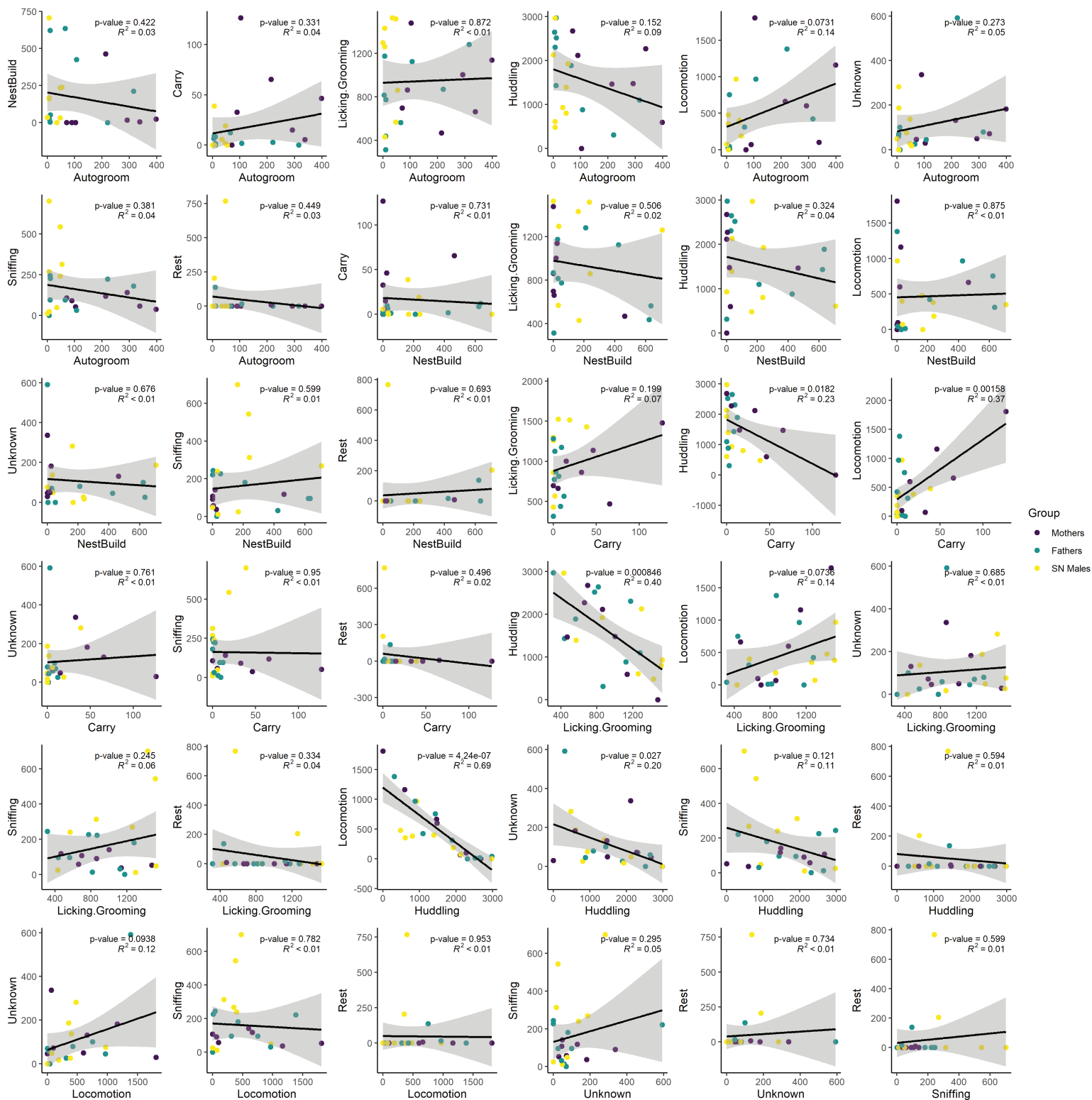

Supplement: Supplementary file 6 — Additional file 6. Relationships between parental behavior test behaviors. For each pair of behavior, a linear model was fit. Each individual point represents a distinct animal, and the shaded area depicts the 95% confidence interval. [file 12864_2022_8912_MOESM6_ESM.pdf]

## A - MPOA

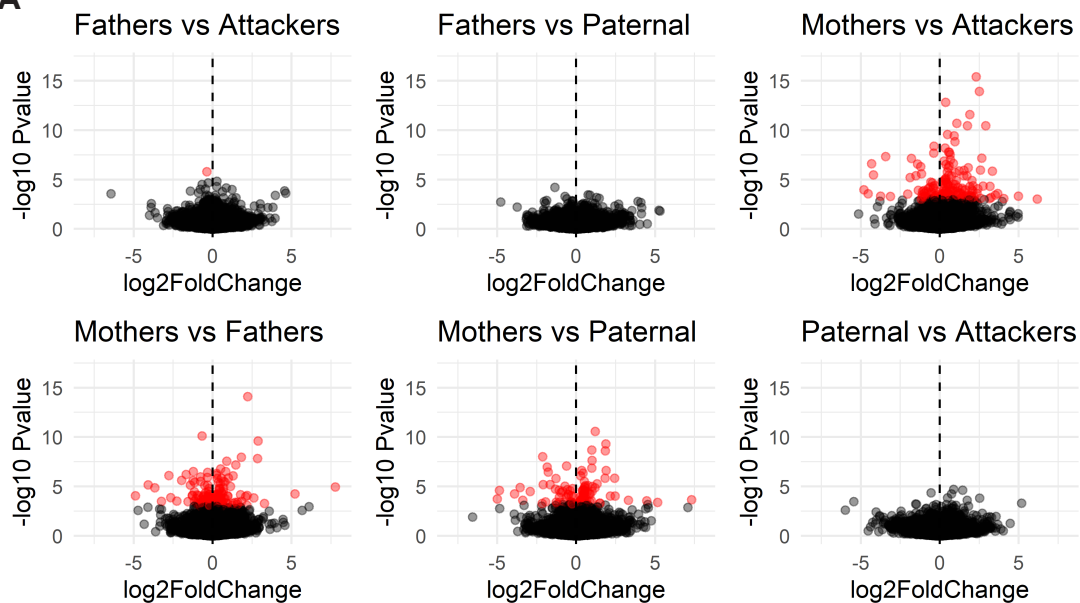

## B - NAc

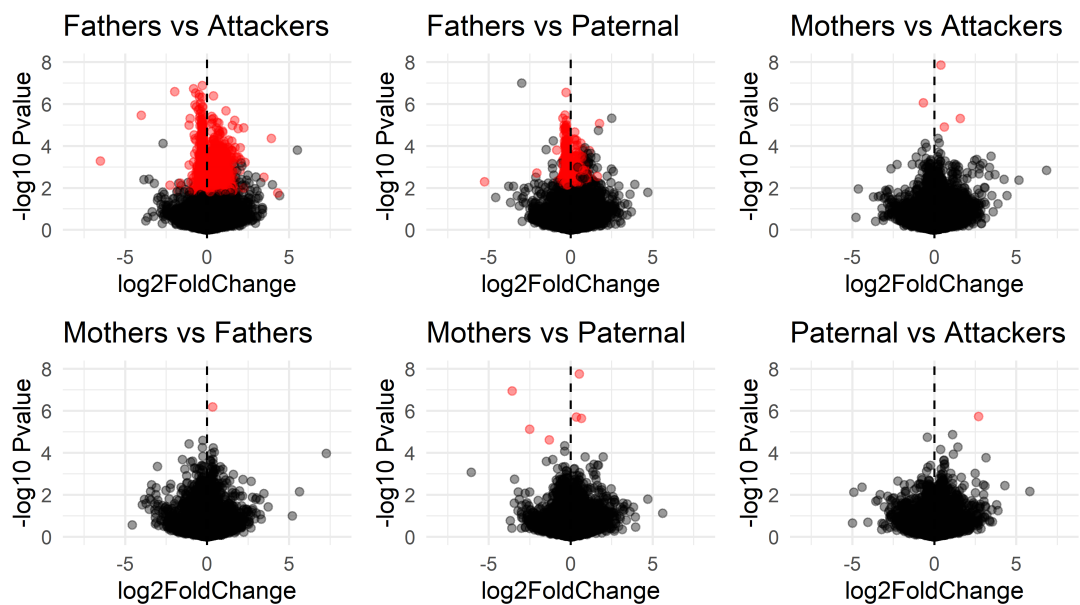

## C - LS

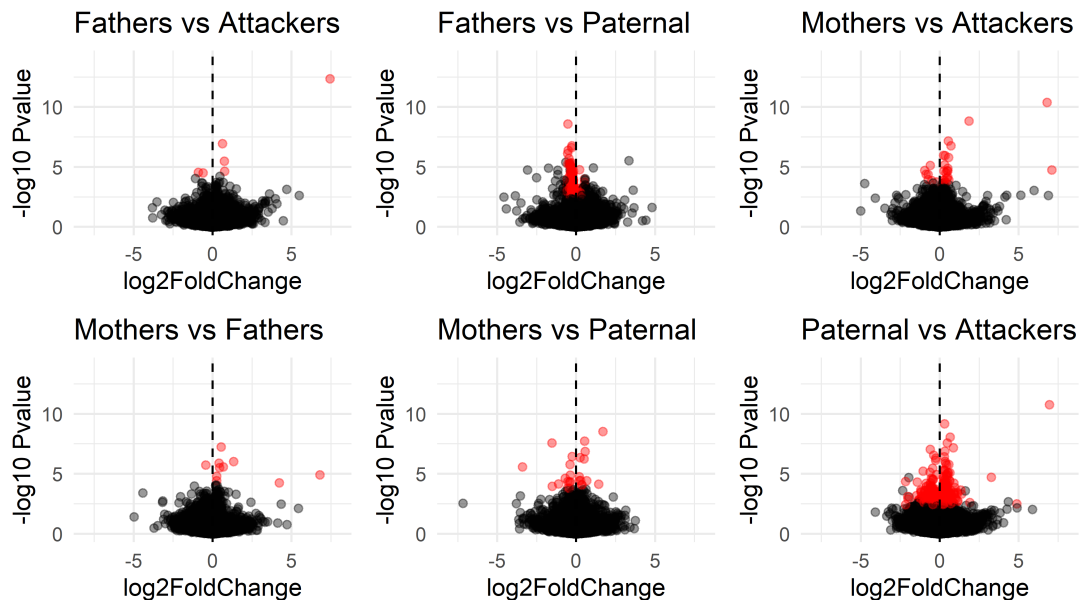

Supplement: Supplementary file 12 — Additional file 12. Volcano plots for the differential expression analysis. These plots depict the log2 fold-change (x-axis) against the -log10 of the uncorrected p-value (y-axis) for each gene in each pairwise comparison in the medial preoptic area (MPOA, A), nucleus accumbens (NAc, B), and lateral septum (LS, C). Differentially expressed genes are depicted in red. [file 12864_2022_8912_MOESM12_ESM.pdf]

## A Biological Processes

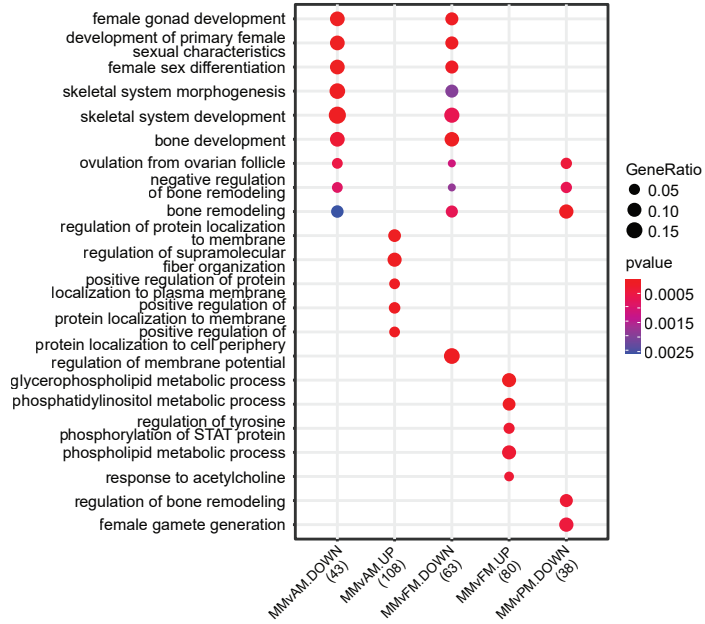

## B Cellular Components

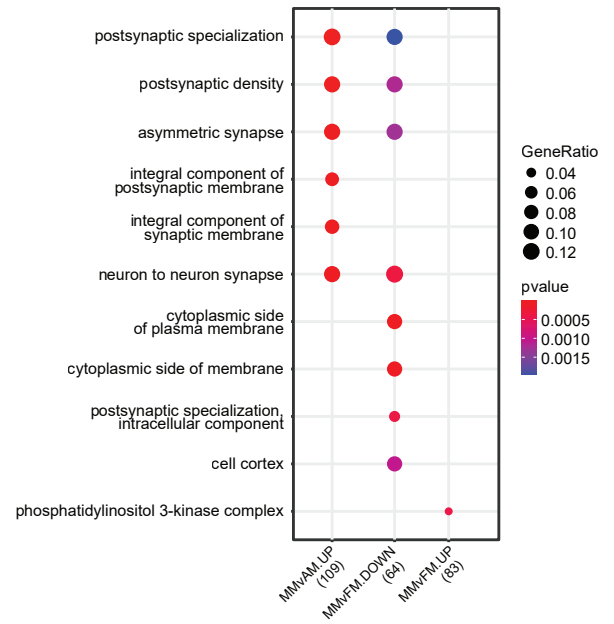

## C Molecular Functions

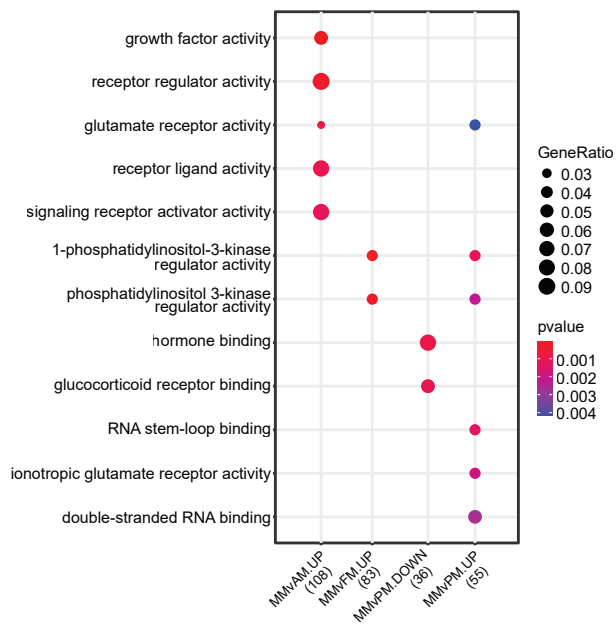

Supplement: Supplementary file 13 — Additional file 13. Functional enrichment of genes differentially expressed in the medial preoptic area (MPOA). For each pairwise comparison, the functional enrichments in gene ontologies of the biological processes (A), cellular components (B), and molecular functions (C) categories were tested for the differentially expressed genes up- or down-regulated (UP, and DOWN, respectively). The labels of pairwise comparisons were coded in two letters: the first one represents the phenotype (M: mothers, F: fathers, P: paternal males, A: attackers), whereas the second one represents the structure (M: MPOA). For instance, MMvFM refers to the comparison between the mothers and fathers in the MPOA. [file 12864_2022_8912_MOESM13_ESM.pdf]

**A**

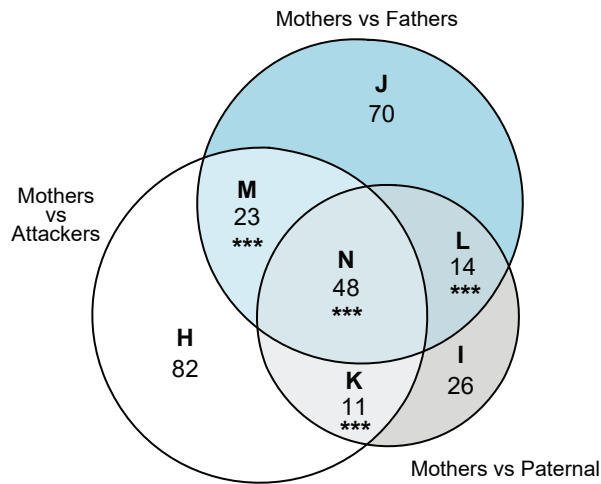

**B**

Biological Processes

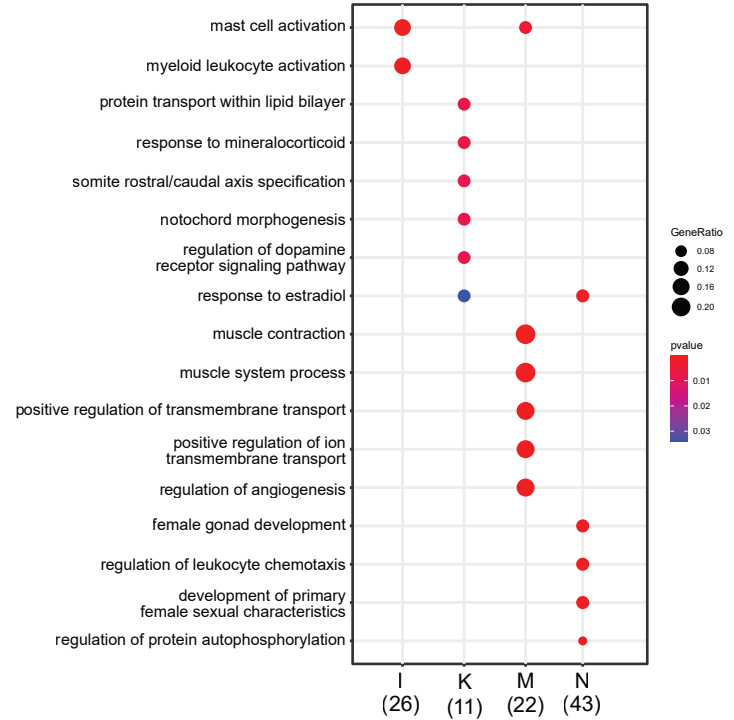

**C**

Cellular Components

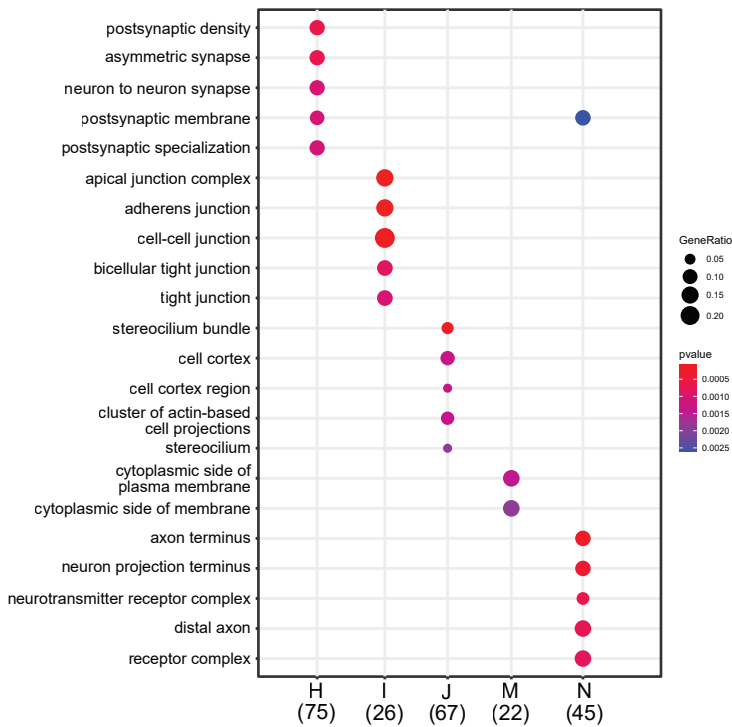

**D**

Molecular Functions

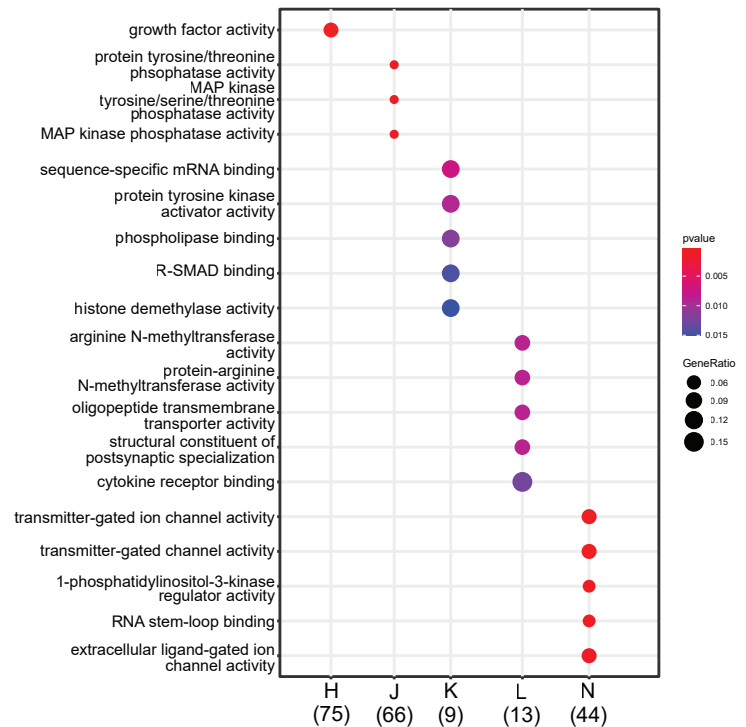

Supplement: Supplementary file 15 — Additional file 15. Functional enrichment of overlapping and distinct sets of differentially expressed genes in the medial preoptic area (MPOA). In (A), the number of differentially expressed genes overlapping or distinct between all sets of sexually biased comparisons in the MPOA is depicted. For each set, the functional enrichments in gene ontologies of the biological processes (B), cellular components (C), and molecular functions (D) categories are displayed. ***p < 0.001, hypergeometric test for overlaps between two or three sets of genes. [file 12864_2022_8912_MOESM15_ESM.pdf]

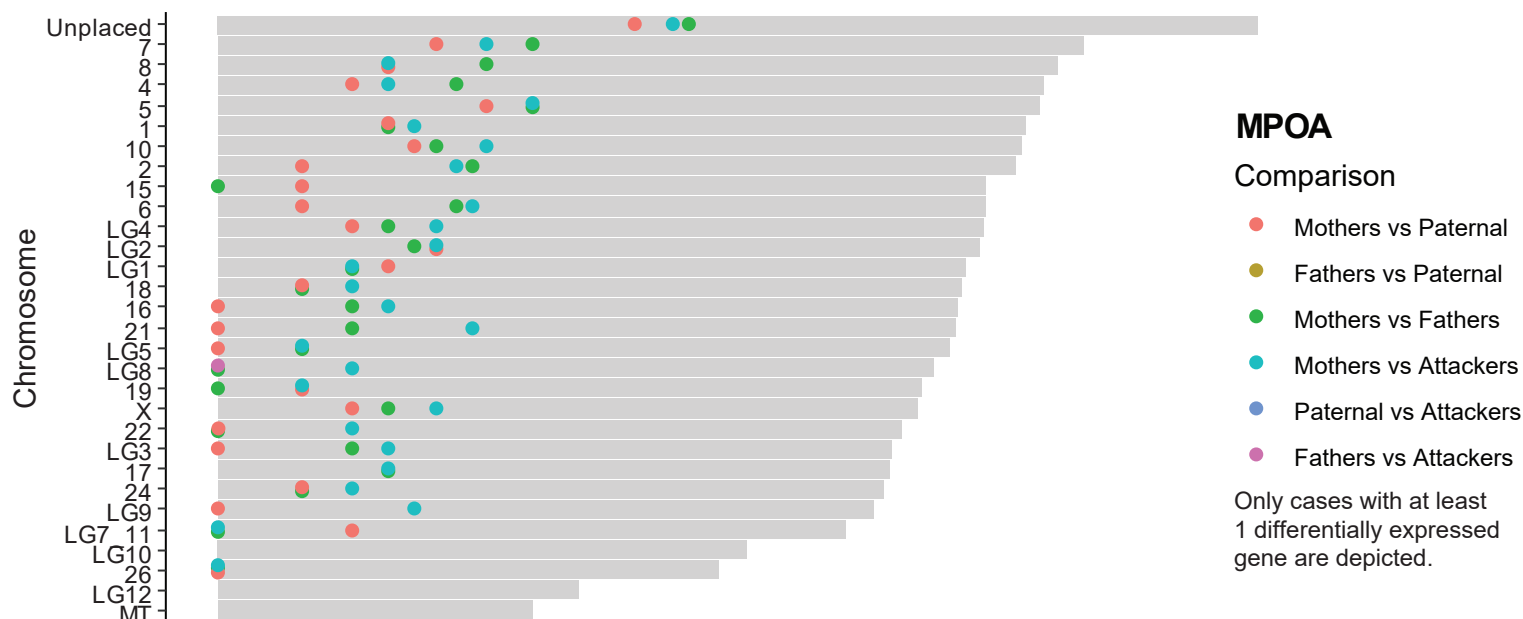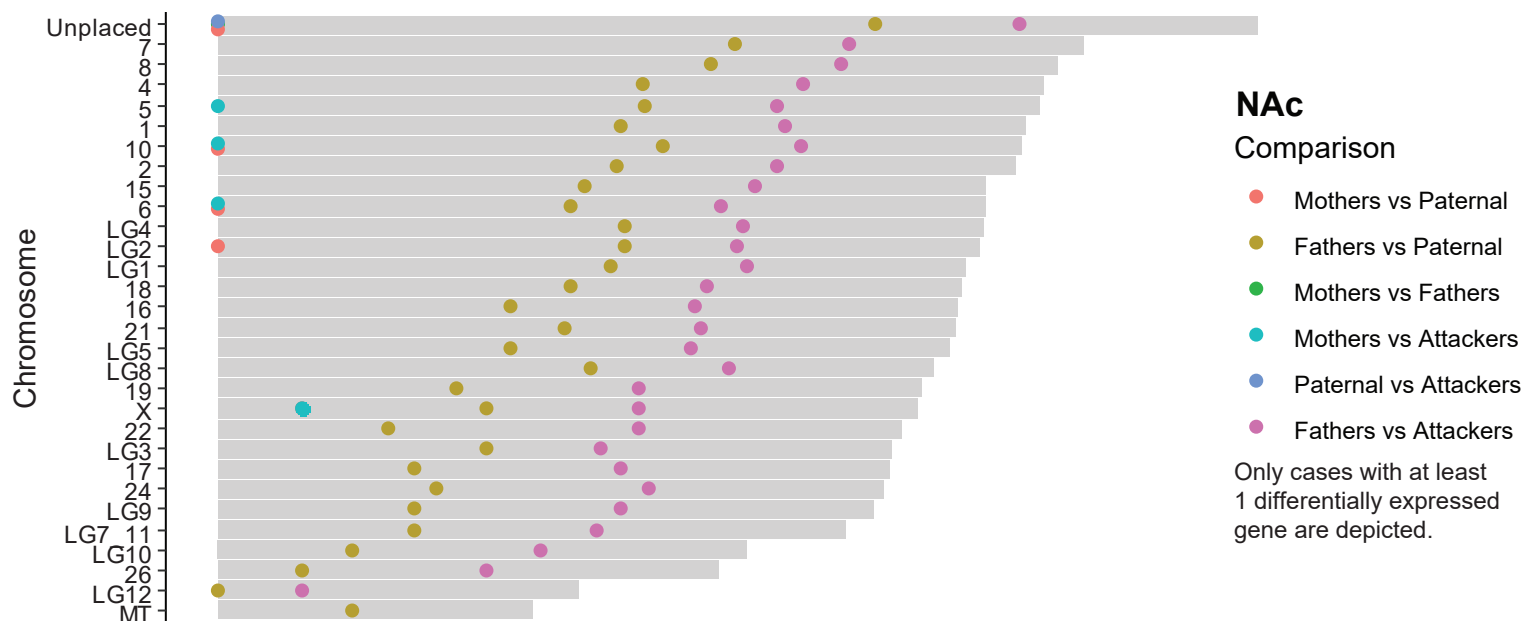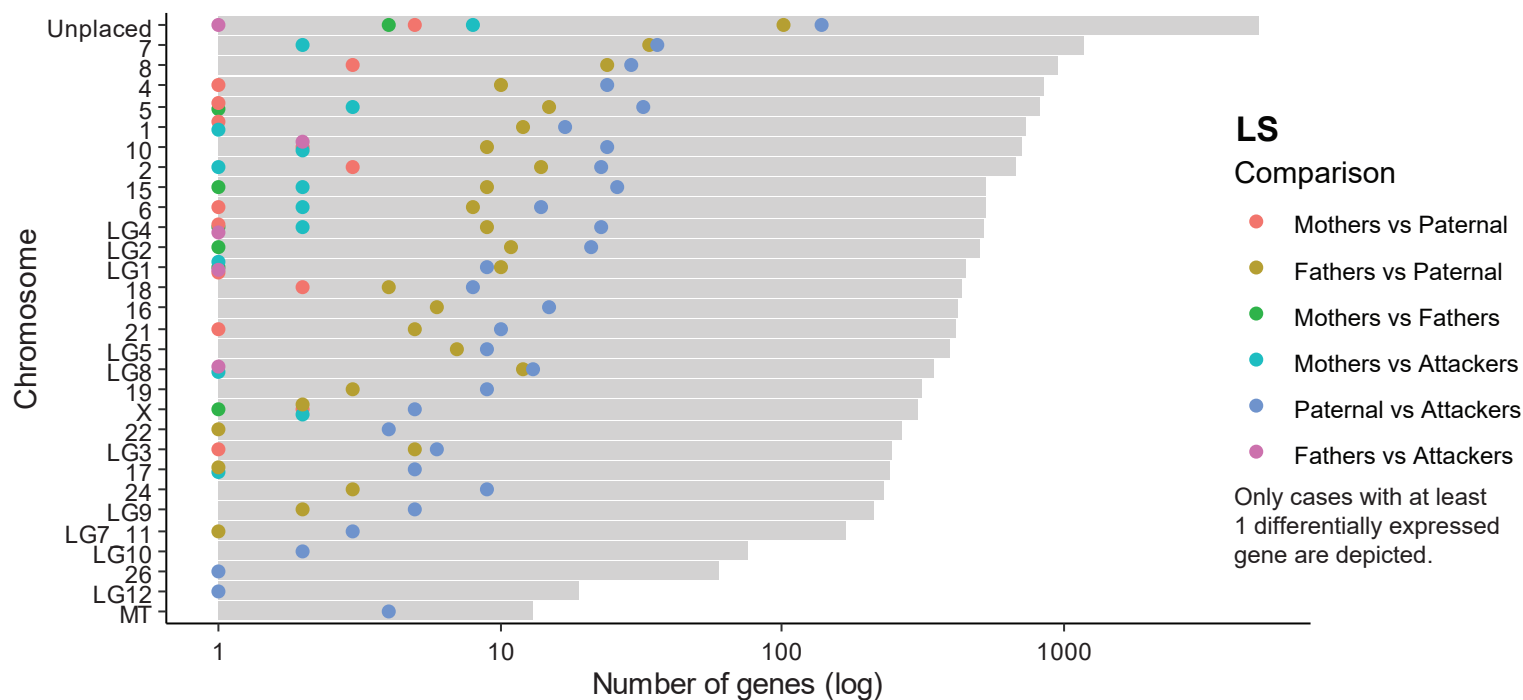

Supplement: Supplementary file 16 — Additional file 16. Representation of the number of genes differentially expressed in the medial preoptic area (MPOA, top), nucleus accumbens (NAc, middle), and lateral septum (LS, bottom) across all chromosomes and linkage groups in the prairie vole assembly. Only cases with at least one differentially expressed gene are depicted. [file 12864_2022_8912_MOESM16_ESM.pdf]

## A Biological Processes

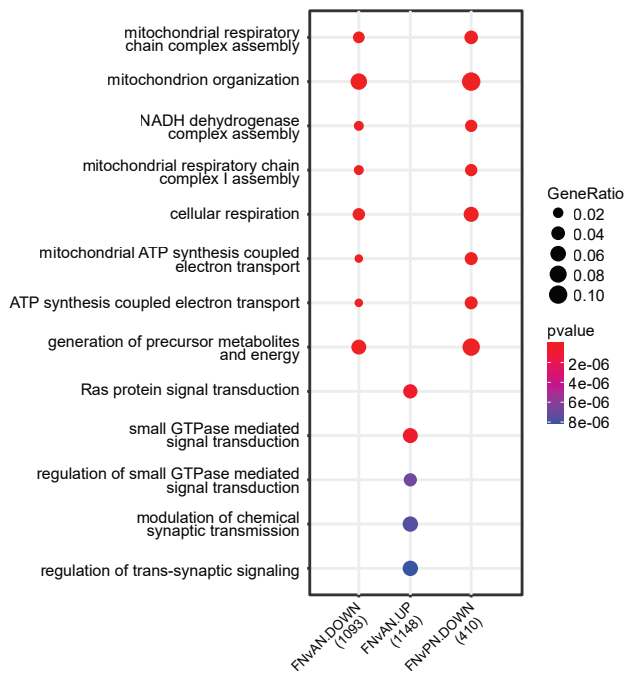

## B Cellular Components

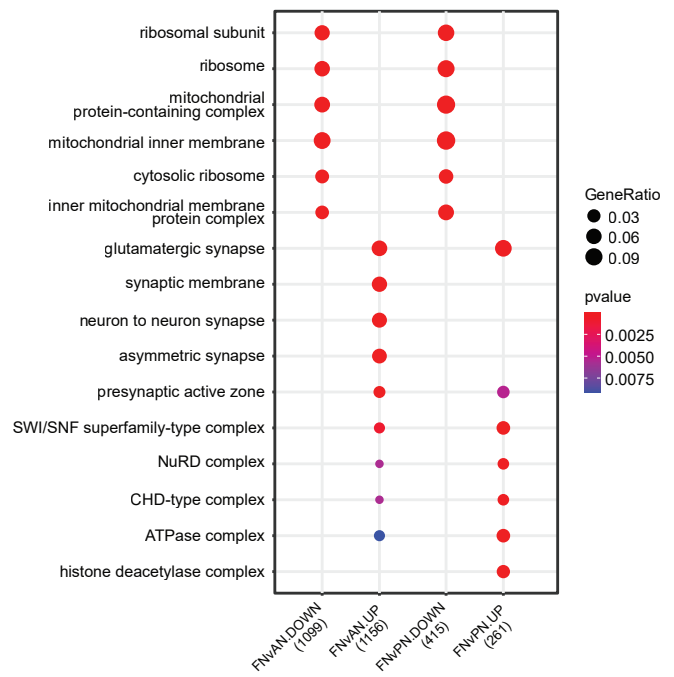

## C Molecular Functions

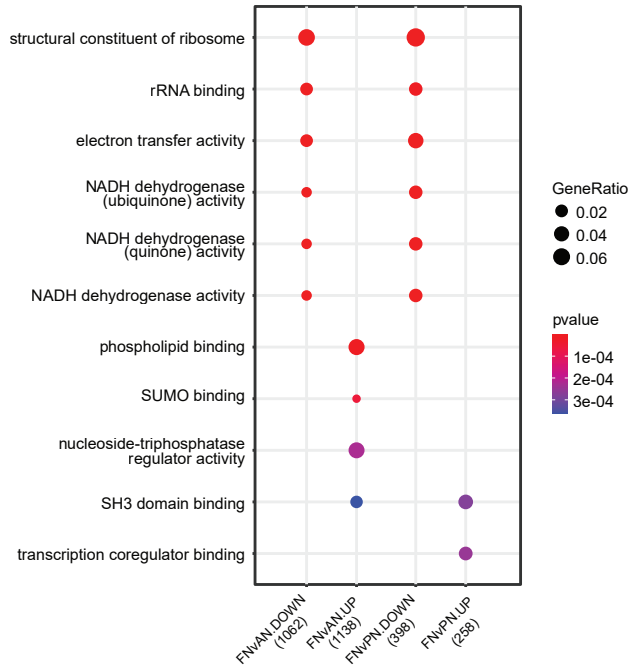

## D Pathways

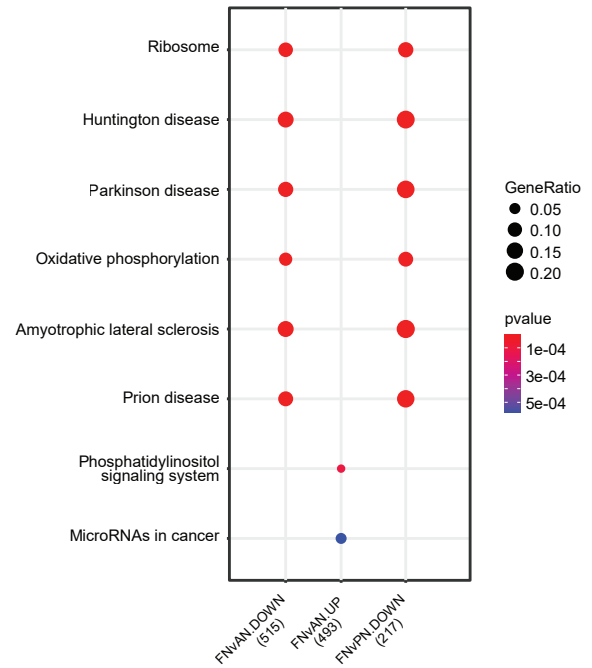

Supplement: Supplementary file 17 — Additional file 17. Functional enrichment of genes differentially expressed in the nucleus accumbens (NAc). For each pairwise comparison, the functional enrichments in gene ontologies of the biological processes (A), cellular components (B), and molecular functions (C) categories as well as in pathways from the Kyoto Encyclopedia of Genes and Genomes (KEGG, D) were tested for the differentially expressed genes up- or down-regulated (UP, and DOWN, respectively). The labels of pairwise comparisons were coded in two letters: the first one represents the phenotype (M: mothers, F: fathers, P: paternal males, A: attackers), whereas the second one represents the structure (N: NAc). For instance, FNvPN refers to the comparison between fathers and paternal males in the NAc. [file 12864_2022_8912_MOESM17_ESM.pdf]

**A** Biological Processes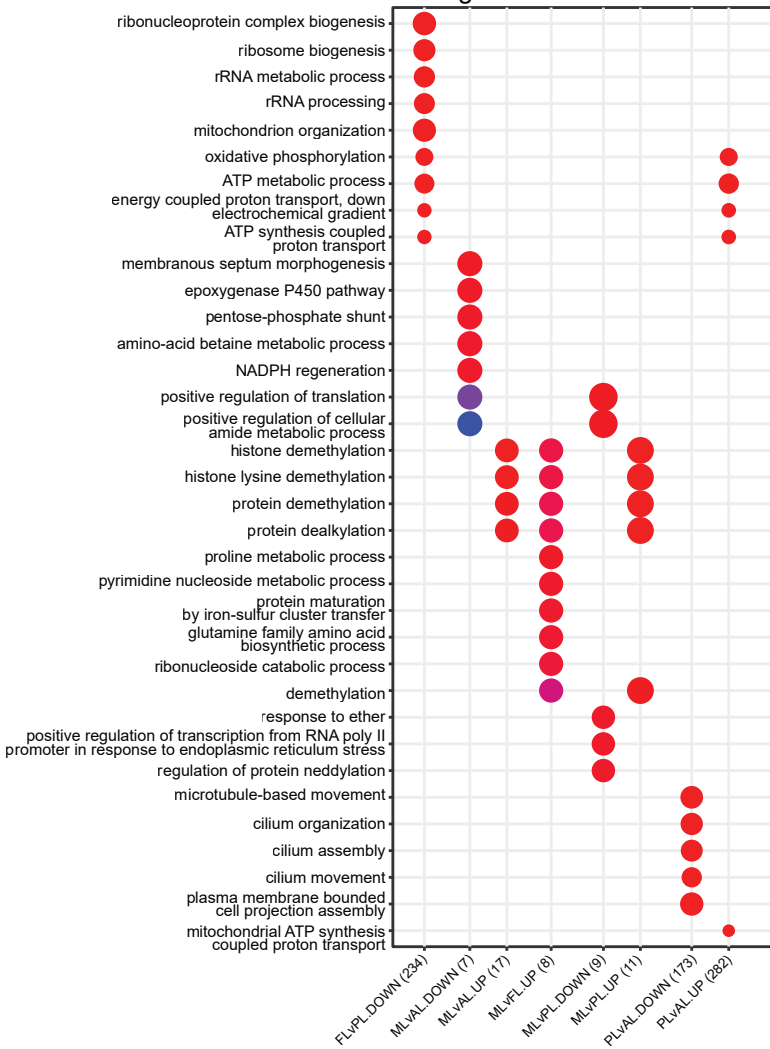**B** Cellular Components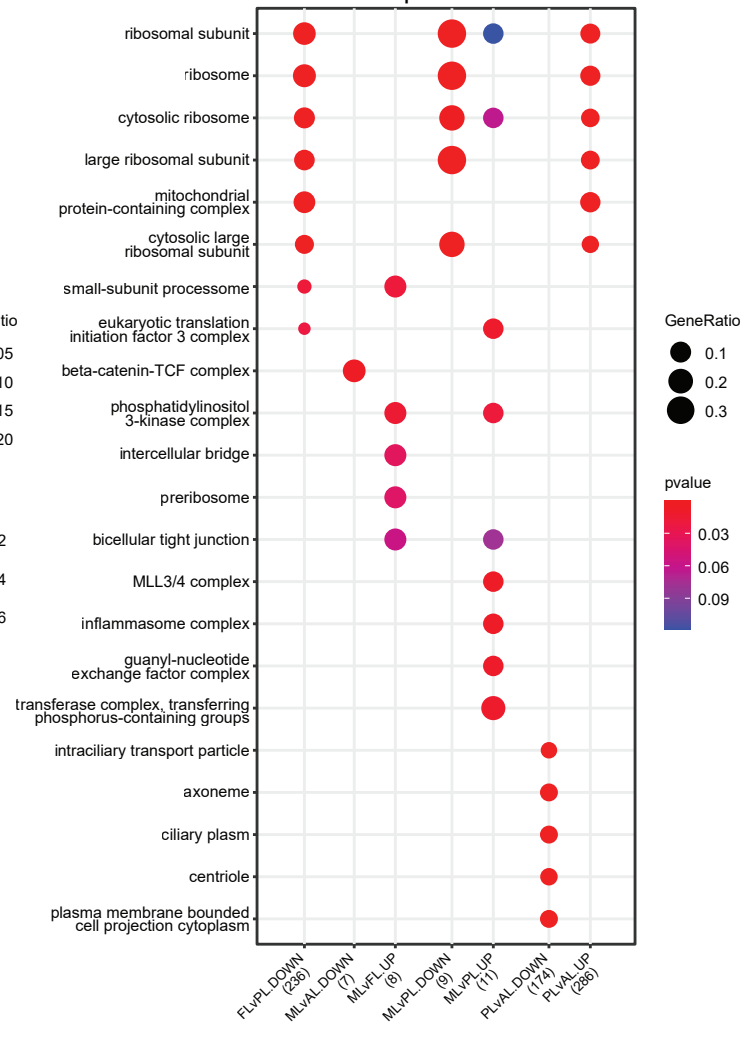**C** Molecular Functions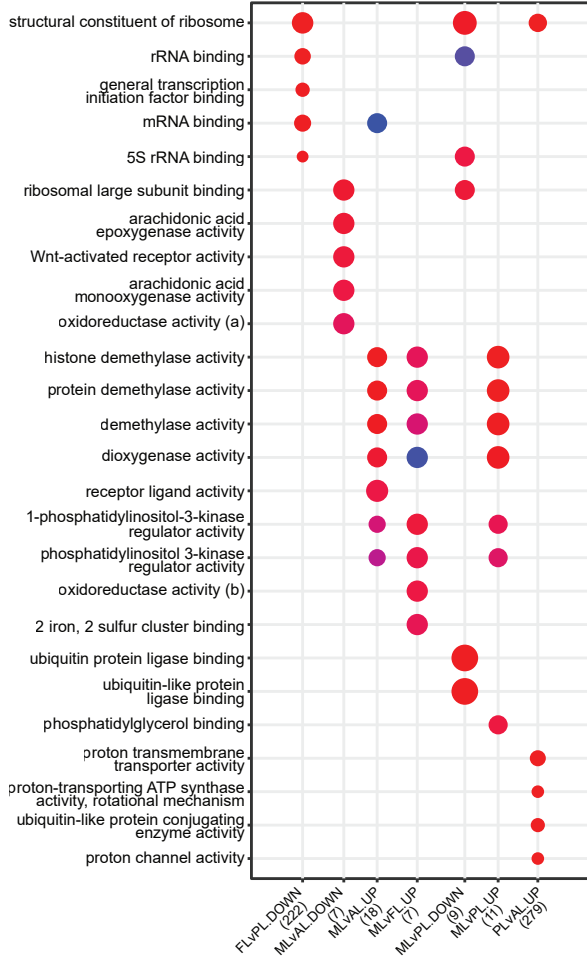**D** Pathways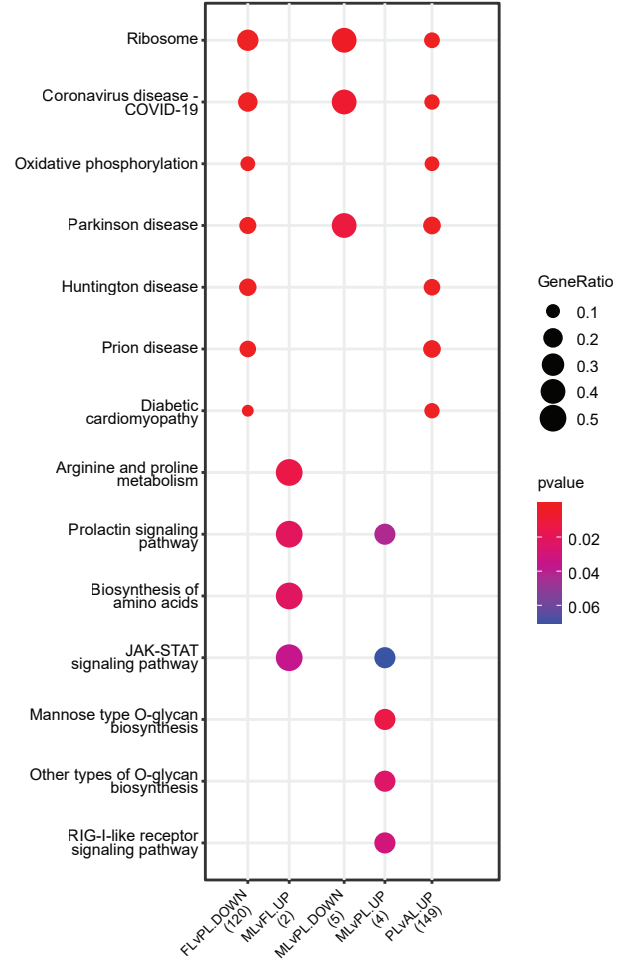

Supplement: Supplementary file 18 — Additional file 18. Functional enrichment of genes differentially expressed in the lateral septum (LS). For each pairwise comparison, the functional enrichments in gene ontologies of the biological processes (A), cellular components (B), and molecular functions (C) categories as well as in pathways from the Kyoto Encyclopedia of Genes and Genomes (KEGG, D) were tested for the differentially expressed genes up- or down-regulated (UP, and DOWN, respectively). The labels of pairwise comparisons were coded in two letters: the first one represents the phenotype (M: mothers, F: fathers, P: paternal males, A: attackers), whereas the second one represents the structure (L: LS). For instance, PLvAL refers to the comparison between the Paternal and Attackers phenotypes in the LS. [file 12864_2022_8912_MOESM18_ESM.pdf]

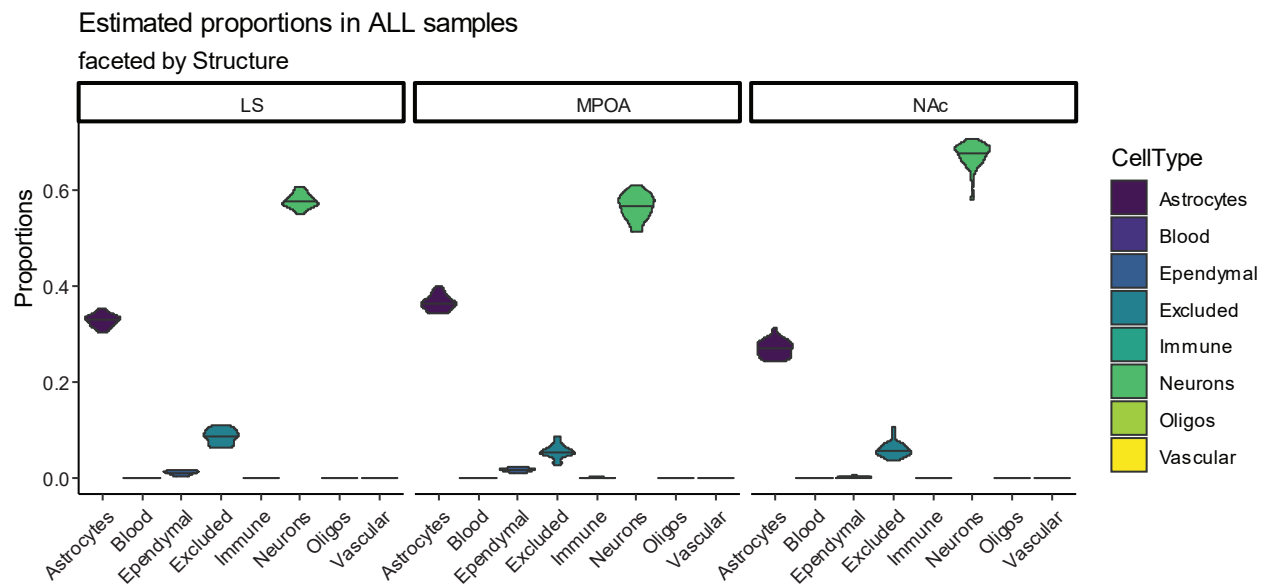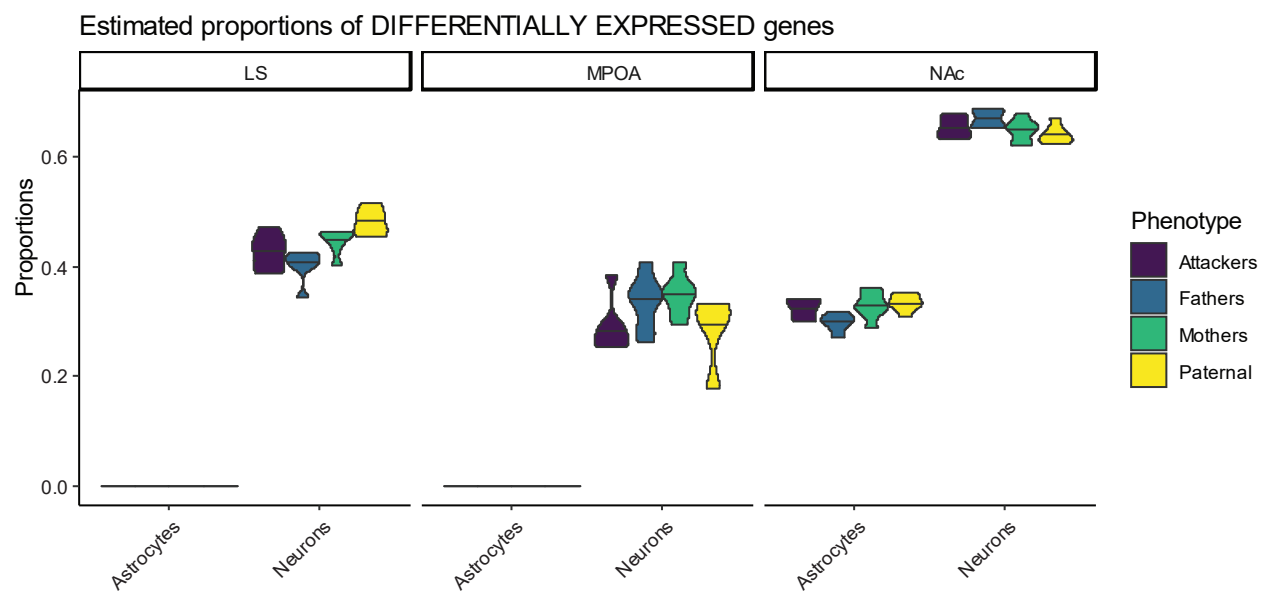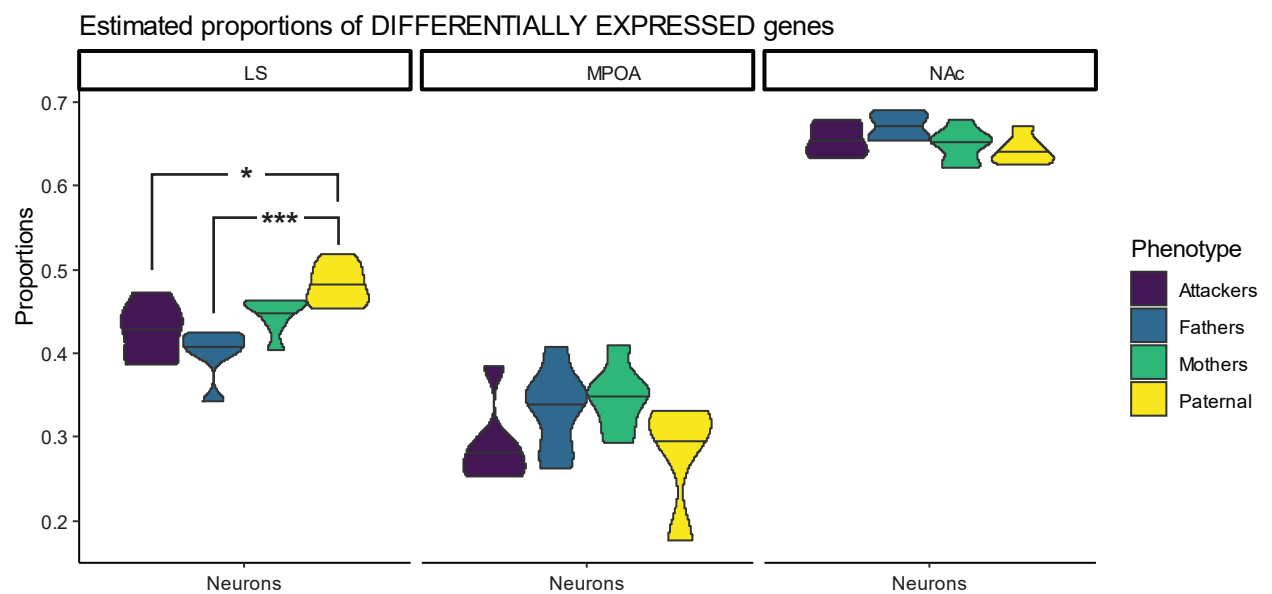

Supplement: Supplementary file 19 — Additional file 19. Estimated cell type proportions. The proportions of various cell types were estimated in our dataset using a publicly-available single-cell RNA sequencing dataset. While panel (A) shows the estimated proportions for all genes detected in our study, panels (B) and (C) depict the estimated proportion of the “Astrocytes” and “Neurons” cell types, or only “Neurons”, respectively, in genes differentially expressed in the given structure. [file 12864_2022_8912_MOESM19_ESM.pdf]

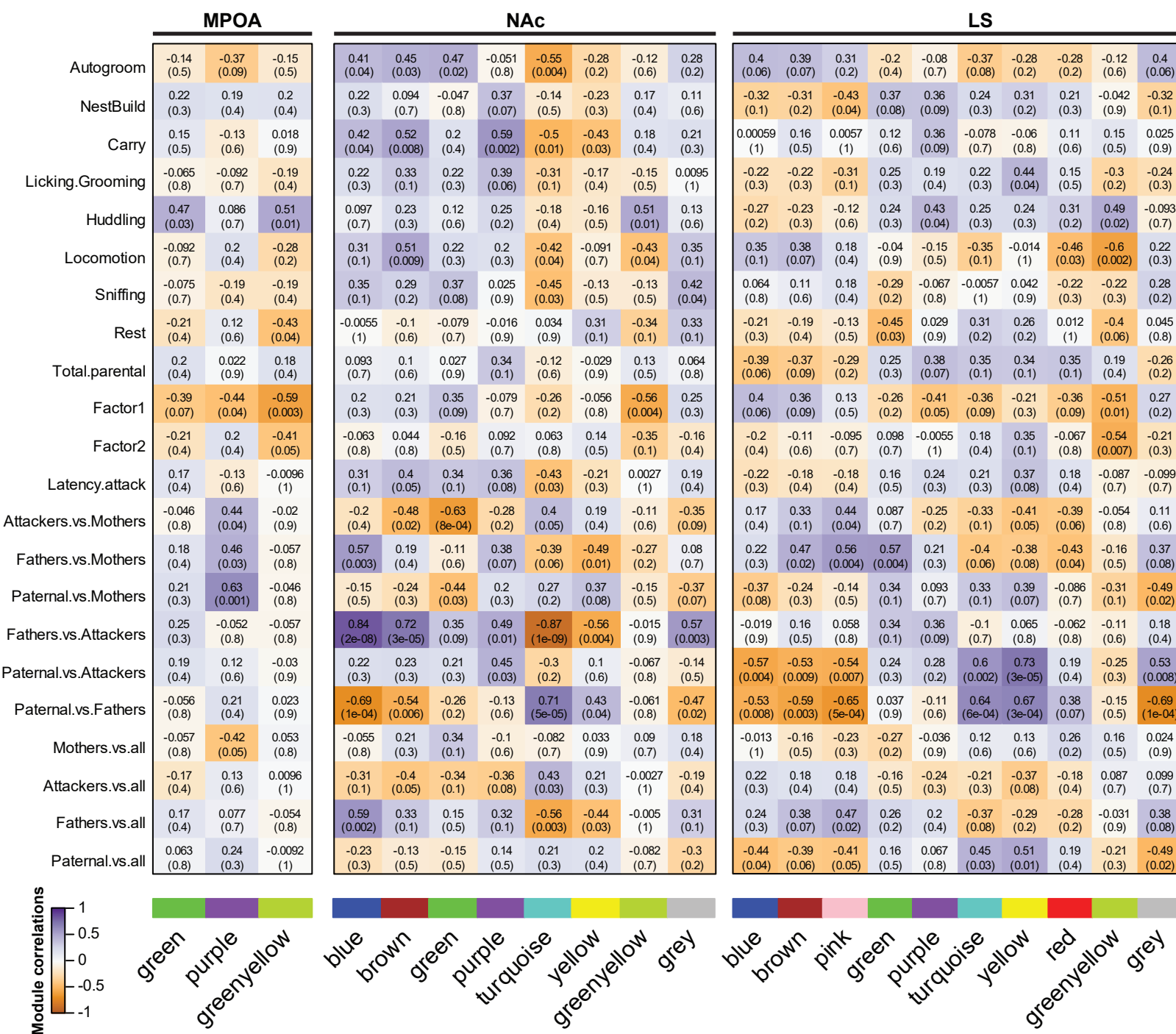

Supplement: Supplementary file 22 — Additional file 22. Structure-specific associations of gene coexpression modules with parental behaviors. The correlation of each co-expression module from the weighted gene coexpression network analysis with behavioral traits (behaviors scored during parental behavior test and phenotype status) is depicted for each structure. The correlation value is detailed alongside its corresponding p-value in parentheses. Note that within each structure, only modules with at least one significant association are depicted. MPOA: medial preoptic area, NAc: nucleus accumbens, LS: lateral septum. [file 12864_2022_8912_MOESM22_ESM.pdf]

WGCNA - Biological Processes

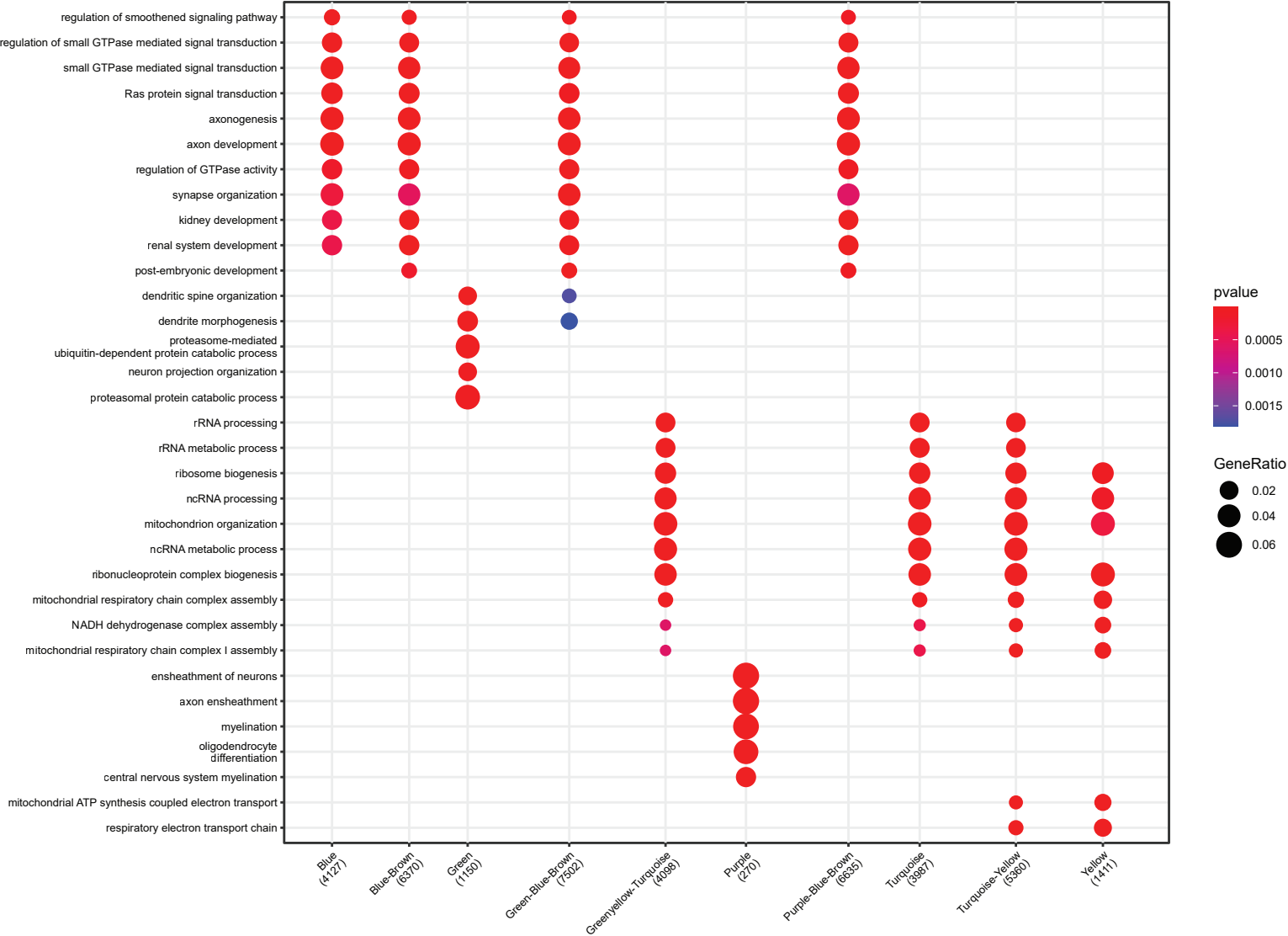

Supplement: Supplementary file 24 — Additional file 24. Functional enrichment of gene ontologies of the biological processes category in gene co-expression modules derived from the weighted gene coexpression network analysis (WGCNA). [file 12864_2022_8912_MOESM24_ESM.pdf]

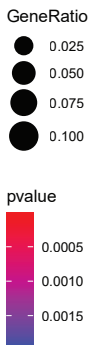

Supplement: Supplementary file 27 — Additional file 27. Functional enrichment of pathways from the Kyoto Encyclopedia of Genes and Genomes (KEGG) in gene co-expression modules derived from the weighted gene coexpression network analysis (WGCNA). [file 12864_2022_8912_MOESM27_ESM.pdf]

**A - Plate 15**

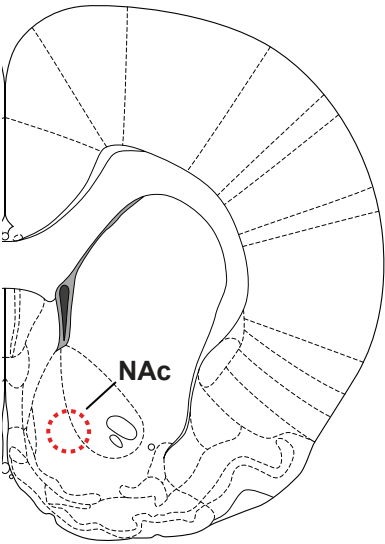

**B - Plate 25**

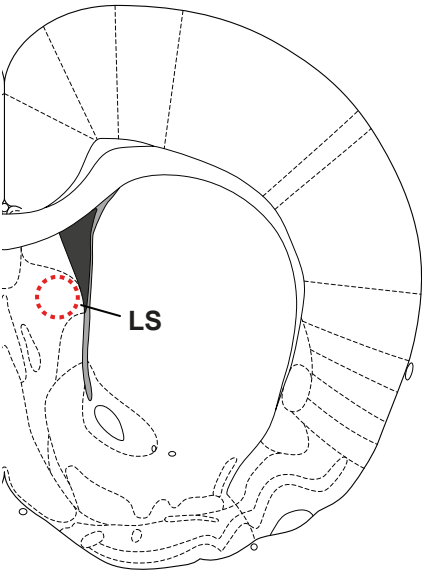

**C - Plate 35**

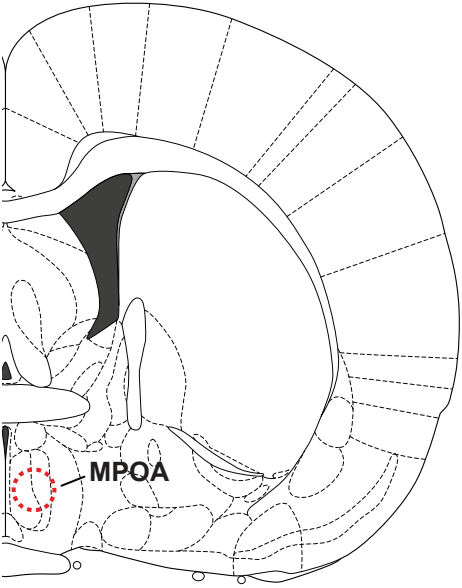

Supplement: Supplementary file 28 — Additional file 28. Representative location of tissue punches collection. As no atlas in stereotaxic coordinates exists for the prairie vole brain, representative plates from the rat brain atlas [95] are depicted. Tissue punches were taken from sections ranging from plates 12–18 for the nucleus accumbens (NAc, A), plates 19–27 for the lateral septum (LS, B), and plates 32–40 for the medial preoptic area (MPOA, C). [file 12864_2022_8912_MOESM28_ESM.pdf]
