# Supplementary material for: Transcriptomic analysis of paternal behaviors in prairie voles
Source: BMC Genomics. 2022 Oct 1;23:679. doi: 10.1186/s12864-022-08912-y (PMC9526941; doi:10.1186/s12864-022-08912-y)
Supplement: Supplementary file 2 — Additional file 2. Analysis of variance (ANOVA) results for all behaviors scored during the parental behavior test. [file 12864_2022_8912_MOESM2_ESM.pdf]

Behavior	term	sumsq	df	statistic	p.value	Eta2
Autogroom	Group	139722125700	2	7.04	0.01	0.40
Carry	Group	6231359453	2	4.97	0.02	0.32
Sniffing	Group	144498675428	2	2.91	0.08	0.22
Licking.Grooming	Group	381340307524	2	1.38	0.27	0.12
Rest	Group	67396595977	2	1.33	0.28	0.11
NestBuild	Group	97359680868	2	0.92	0.41	0.08
total.parental	Group	621745608192	2	0.77	0.47	0.07
Locomotion	Group	291397219229	2	0.58	0.57	0.05
Huddling	Group	649228224387	2	0.39	0.68	0.04
