# Supplementary material for: Transcriptomic analysis of paternal behaviors in prairie voles
Source: BMC Genomics. 2022 Oct 1;23:679. doi: 10.1186/s12864-022-08912-y (PMC9526941; doi:10.1186/s12864-022-08912-y)
Supplement: Supplementary file 5 — Additional file 5. Analysis of variance (ANOVA) results for behavioral bouts analysis. [file 12864_2022_8912_MOESM5_ESM.pdf]

Behavior	Variable	term	df	sumsq	meansq	statistic	p.value	Eta2
Autogroom	mean	Group	2	948.94	474.47	2.13	0.14	0.17
Autogroom	median	Group	2	201.41	100.71	0.71	0.50	0.06
Carry	mean	Group	2	135.80	67.90	1.73	0.20	0.14
Carry	median	Group	2	143.19	71.60	1.91	0.17	0.15
Huddling	mean	Group	2	7894.69	3947.34	0.34	0.71	0.03
Huddling	median	Group	2	1030.08	515.04	0.10	0.91	0.01
Licking.Grooming	mean	Group	2	587.43	293.71	0.48	0.62	0.04
Licking.Grooming	median	Group	2	1458.77	729.38	1.33	0.29	0.11
Locomotion	mean	Group	2	408.24	204.12	0.25	0.78	0.02
Locomotion	median	Group	2	99.36	49.68	0.25	0.78	0.02
NestBuild	mean	Group	2	812.43	406.22	1.45	0.26	0.12
NestBuild	median	Group	2	1022.51	511.25	2.59	0.10	0.20
Rest	mean	Group	2	2669.11	1334.56	1.19	0.32	0.10
Rest	median	Group	2	1902.11	951.05	1.04	0.37	0.09
Sniffing	mean	Group	2	21.45	10.72	0.05	0.95	0.00
Sniffing	median	Group	2	202.98	101.49	0.40	0.67	0.04
total.parental	mean	Group	2	948456.72	474228.36	0.79	0.47	0.07
total.parental	median	Group	2	715353.41	357676.71	0.57	0.58	0.05
