# Supplementary material for: Transcriptomic analysis of paternal behaviors in prairie voles
Source: BMC Genomics. 2022 Oct 1;23:679. doi: 10.1186/s12864-022-08912-y (PMC9526941; doi:10.1186/s12864-022-08912-y)
Supplement: Supplementary file 8 — Additional file 8. Comparisons of slopes for linear regressions between behaviors scored during the parental behaviors test. Slopes (a) and their differences between groups (b) are listed for all linear regressions with a significant interaction with Group (see Additional file 7). [file 12864_2022_8912_MOESM8_ESM.pdf]

a. Linear regression relationships slopes

Var_X	Var_Y	Group	trend	std.error	df	statistic	p.value
NestBuild	Rest	Mothers	55.49	26.38	18	2.10	0.05
NestBuild	Rest	Fathers	3.54	1.57	18	2.26	0.04
NestBuild	Rest	Paternal	-0.04	0.28	18	-0.15	0.88
Carry	Huddling	Mothers	-0.04	0.01	18	-5.99	0.00
Carry	Huddling	Fathers	0.00	0.01	18	0.09	0.93
Carry	Huddling	Paternal	-0.01	0.01	18	-1.42	0.17
Carry	Locomotion	Mothers	0.06	0.01	18	6.28	0.00
Carry	Locomotion	Fathers	0.00	0.01	18	-0.11	0.91
Carry	Locomotion	Paternal	0.01	0.02	18	0.75	0.46
Locomotion	Huddling	Mothers	-0.69	0.08	18	-8.25	0.00
Locomotion	Huddling	Fathers	-0.51	0.08	18	-6.72	0.00
Locomotion	Huddling	Paternal	-0.24	0.08	18	-2.81	0.01
Unknown	Locomotion	Mothers	-0.05	0.07	18	-0.64	0.53
Unknown	Locomotion	Fathers	0.28	0.08	18	3.40	0.00
Unknown	Locomotion	Paternal	0.11	0.15	18	0.77	0.45
Sniffing	Carry	Mothers	-0.32	0.93	18	-0.34	0.73
Sniffing	Carry	Fathers	-13.27	7.77	18	-1.71	0.10
Sniffing	Carry	Paternal	15.03	2.70	18	5.56	0.00
Sniffing	Huddling	Mothers	0.02	0.06	18	0.31	0.76
Sniffing	Huddling	Fathers	-0.01	0.05	18	-0.10	0.92
Sniffing	Huddling	Paternal	-0.19	0.06	18	-3.20	0.00

b. Linear regression relationships slopes comparisons

Var_X	Var_Y	term	contrast	null.value	estimate	std.error	df	statistic	adj.p.value
NestBuild	Rest	Group	Mothers - Fathers	0	51.95	26.43	18	1.97	0.15
NestBuild	Rest	Group	Mothers - Paternal	0	55.53	26.38	18	2.10	0.12
NestBuild	Rest	Group	Fathers - Paternal	0	3.58	1.59	18	2.25	0.09
Carry	Huddling	Group	Mothers - Fathers	0	-0.04	0.01	18	-4.50	0.00
Carry	Huddling	Group	Mothers - Paternal	0	-0.03	0.01	18	-3.21	0.01
Carry	Huddling	Group	Fathers - Paternal	0	0.01	0.01	18	1.12	0.52
Carry	Locomotion	Group	Mothers - Fathers	0	0.06	0.01	18	4.19	0.00
Carry	Locomotion	Group	Mothers - Paternal	0	0.04	0.02	18	2.07	0.12
Carry	Locomotion	Group	Fathers - Paternal	0	-0.02	0.02	18	-0.71	0.76
Locomotion	Huddling	Group	Mothers - Fathers	0	-0.18	0.11	18	-1.58	0.28
Locomotion	Huddling	Group	Mothers - Paternal	0	-0.45	0.12	18	-3.80	0.00
Locomotion	Huddling	Group	Fathers - Paternal	0	-0.27	0.11	18	-2.41	0.07
Unknown	Locomotion	Group	Mothers - Fathers	0	-0.33	0.11	18	-2.99	0.02
Unknown	Locomotion	Group	Mothers - Paternal	0	-0.16	0.16	18	-0.97	0.60
Unknown	Locomotion	Group	Fathers - Paternal	0	0.17	0.17	18	0.99	0.59
Sniffing	Carry	Group	Mothers - Fathers	0	12.95	7.82	18	1.66	0.25
Sniffing	Carry	Group	Mothers - Paternal	0	-15.35	2.86	18	-5.37	0.00
Sniffing	Carry	Group	Fathers - Paternal	0	-28.30	8.23	18	-3.44	0.01
Sniffing	Huddling	Group	Mothers - Fathers	0	0.02	0.08	18	0.30	0.95
Sniffing	Huddling	Group	Mothers - Paternal	0	0.21	0.08	18	2.50	0.06
Sniffing	Huddling	Group	Fathers - Paternal	0	0.18	0.08	18	2.32	0.08
