# Supplementary material for: Transcriptomic analysis of paternal behaviors in prairie voles
Source: BMC Genomics. 2022 Oct 1;23:679. doi: 10.1186/s12864-022-08912-y (PMC9526941; doi:10.1186/s12864-022-08912-y)
Supplement: Supplementary file 20 — Additional file 20. Analysis of variance (ANOVA) results for the estimated proportions of the “Neurons” cell type in the genes differentially expressed in the medial preoptic area (MPOA), nucleus accumbens (NAc), and lateral septum (LS).) [file 12864_2022_8912_MOESM20_ESM.pdf]

| Structure | term | sumsq | df | statistic | p.value | Eta2 |
|---|---|---|---|---|---|---|
| LS | Phenotype | 0.0208 | 3 | 8.80 | 0.01 | 0.58 |
| MPOA | Phenotype | 0.0173 | 3 | 2.15 | 0.13 | 0.26 |
| NAc | Phenotype | 0.0026 | 3 | 2.59 | 0.08 | 0.28 |
