# Supplementary material for: Transcriptomic analysis of paternal behaviors in prairie voles
Source: BMC Genomics. 2022 Oct 1;23:679. doi: 10.1186/s12864-022-08912-y (PMC9526941; doi:10.1186/s12864-022-08912-y)

WGCNA - Molecular Functions

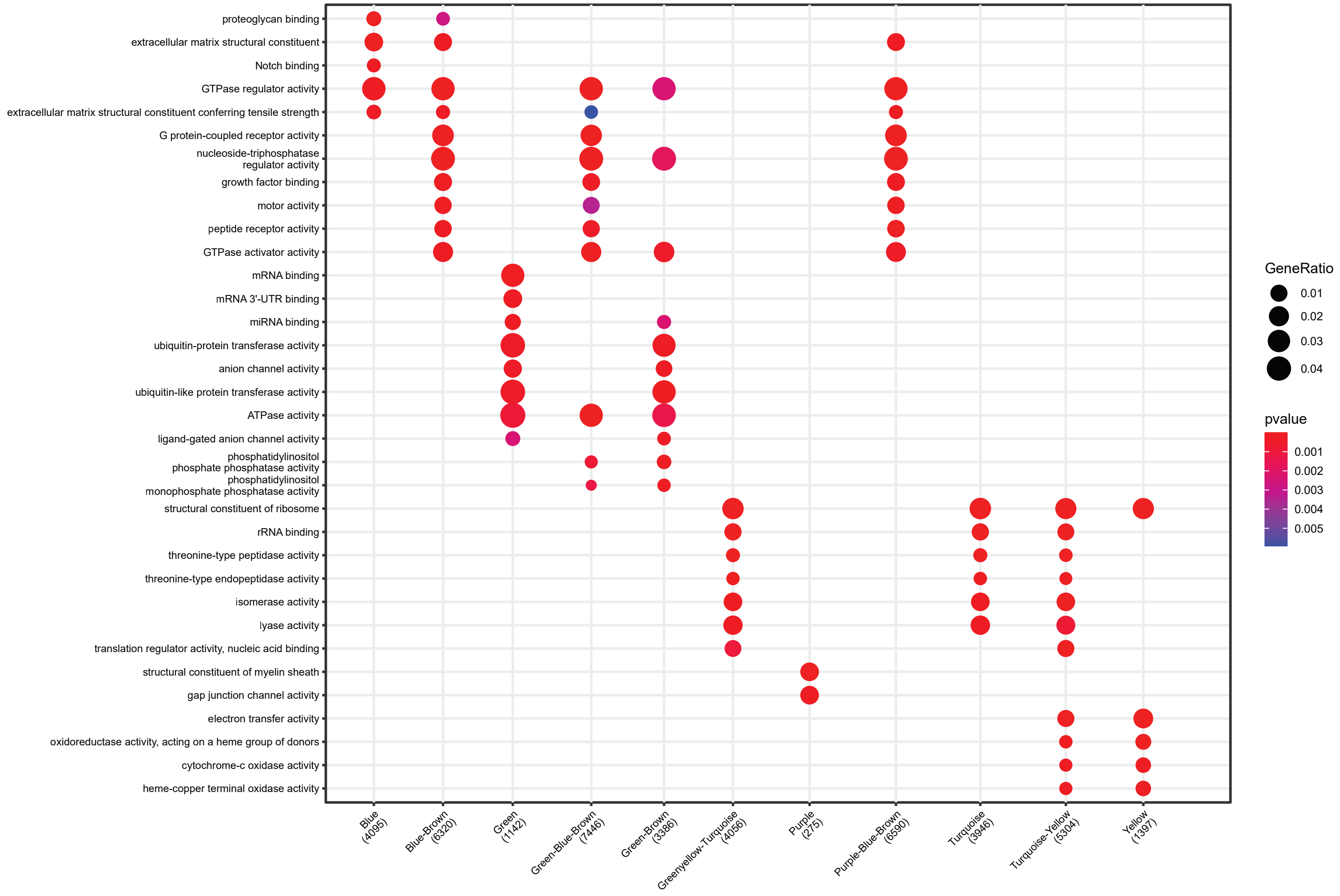

Supplement: Supplementary file 26 — Additional file 26. Functional enrichment of gene ontologies of the molecular functions category in gene co-expression modules derived from the weighted gene coexpression network analysis (WGCNA). [file 12864_2022_8912_MOESM26_ESM.pdf]
